# *Bmi1* controls auditory sensory epithelial cell proliferation through genome-wide H3K27me3 modifications

**DOI:** 10.1186/s13072-025-00642-1

**Published:** 2025-11-19

**Authors:** Xiaoling Lu, Yunzhong Zhang, Ruofei Dai, Kunkun Wang, Fei Lan, Huiqian Yu, Liping Zhao, Renjie Chai, Shan Sun

**Affiliations:** 1 Department of ENT Institute and Otorhinolaryngology, Eye & ENT Hospital, Shanghai Key Laboratory of Gene Editing and Cell Therapy for Rare Diseases, NHC Key Laboratory of Hearing Medicine Research, Fudan University, Shanghai, 200032 People’s Republic of China; 2 Institutes of Biomedical Sciences, Fudan University, Shanghai, 200032 People’s Republic of China; 3 State Key Laboratory of Digital Medical Engineering, Department of Otolaryngology Head and Neck Surgery, Zhongda Hospital, Southeast University, Nanjing, 210096 China; 4 Co-Innovation Center of Neuroregeneration, Nantong University, Nantong, 226001 China; 5 ENT Institute and Otorhinolaryngology Department of Affiliated Eye and ENT Hospital, Fudan University, 83 Fenyang Road, Shanghai, 200031 China

**Keywords:** *Bmi1*, H3K27me3, Histone modification, Inner ear, Cell proliferation, Hearing loss

## Abstract

**Background:**

Bmi1, a key component of the Polycomb repressive complex 1, plays a critical role in regulating gene expression by modulating chromatin structure. Its depletion is known to cause hair cell loss in the neonatal mouse cochlea. This study aimed to investigate the epigenetic mechanisms and transcriptional consequences of Bmi1 depletion in the neonatal auditory sensory epithelium.

**Results:**

Analysis of neonatal Bmi1 knockout mice using H3K27me3 chromatin immunoprecipitation sequencing, assay for transposase-accessible chromatin sequencing, and RNA sequencing revealed significant transcriptional alterations, particularly in genes governing cell proliferation, senescence, and death. *Bmi1* depletion resulted in widespread gene upregulation and increased chromatin accessibility, which correlated with reduced H3K27me3 enrichment. Notably, expression of *Cdkn2c*, a key cell cycle regulator, was significantly upregulated. Inhibition of *Cdkn2c* rescued the proliferative capacity of inner ear epithelial cells in Bmi1 knockout mice.

**Conclusions:**

These findings demonstrate that Bmi1 maintains transcriptional repression and chromatin state in the developing cochlea, primarily through H3K27me3 deposition. Depletion disrupts this control, leading to *Cdkn2c* overexpression and impaired cell proliferation. This identifies *Cdkn2c* and its regulatory pathway as potential therapeutic targets for hearing loss associated with hair cell depletion.

**Supplementary Information:**

The online version contains supplementary material available at 10.1186/s13072-025-00642-1.

## Background

The inner ear hair cells are specialized mechanosensory cells crucial for both auditory and balance functions, and their loss is the leading cause of hearing and balance impairment [1]. While advancements have been made in understanding the signaling pathways and master regulators involved in sensory progenitor cell proliferation and differentiation, the molecular and epigenetic mechanisms governing the formation, maintenance, and regeneration of the auditory system remain poorly understood. This knowledge gap is partly due to the limited availability of inner ear cells and the restricted regenerative capacity of hair cells, particularly in adult organisms.

Polycomb group (PcG) proteins are transcriptional repressors that play a vital role in maintaining appropriate gene expression patterns during development [2]. There are two distinct complexes within the PcGs: the polycomb repressive complex 1 (PRC1) and the polycomb repressive complex 2 (PRC2) [3–5]. The most widely studied polycomb repressive complexes are PRC1 and PRC2. While PRC1 mono-ubiquitylates lysine 119 on histone H2A (H2AK119ub1) through its catalytic subunit RING1A or RING1B ubiquitin ligase, the core subunits of PRC2 catalyze mono-, di-, and trimethylation of lysine 27 on histone H3 [6].

B cell-specific Moloney murine leukemia virus integration site 1 (Bmi1), as a member of the PRC1, plays a critical role in gene regulation through its involvement in histone modifications [7–13]. Loss of *Bmi1* impairs self-renewal in various stem cell populations, including neural, hematopoietic, and intestinal stem cells, resulting in decreased stem cell numbers [11, 14–16]. Conversely, overexpression of *Bmi1* enhances the self-renewal capacity of hematopoietic and neural stem cells [17–21]. Despite these insights, the specific target genes and regulatory pathways through which *Bmi1* functions in the inner ear remain largely unknown.

In our previously studies, we demonstrated that the loss of *Bmi1* has a significant impact on supporting cell proliferation in the neonatal mouse organ of Corti (OC) following neomycin-induced damage [22]. *Bmi1* deficiency impairs the sphere-forming capacity of supporting cells in the OC and reduces the number of sensory epithelial cells [22]. Given Bmi1’s well-established role in epigenetics, we hypothesized that its function in the auditory sensory epithelium is mediated by epigenetic regulation of gene transcription, which could influence hair cell regeneration.

To test this hypothesis, we investigated the impact of *Bmi1* deficiency on the proliferation of auditory sensory epithelium in neonatal mice. We employed ChIP-seq to examine H3K27me3 modifications, ATAC-seq to assess chromatin accessibility, and RNA-seq to analyze transcriptional changes in *Bmi1*^−/−^ auditory sensory epithelial cells. Our study revealed significant transcriptional alterations, a marked reduction in H3K27me3 enrichment, and increased chromatin accessibility, all of which were associated with elevated gene expression including *Cdkn2c*. These findings provide new insights into the epigenetic mechanisms by which *Bmi1* influences auditory cell fate, shedding light on its role in auditory organ development and tissue morphogenesis.

## Methods

### Experimental model details

#### Animals

*Bmi1*^+/−^ mice (FVB.129P2-Bmi1^tm1Brn^/MvlJ, Strain #:024584) were backcrossed 10–12 times into a C57BL/6 J background and then were mated to generate *Bmi1*^−/−^, *Bmi1*^±^, and WT mice (littermates) [23]. All animal experiments were approved by the Animal Care and Use Committee of Fudan University and were conducted in accordance with the National Institutes of Health Guide for the Care and Use of Laboratory Animals (No. 202405024Z). The initiation of this study dates back to 2016. Due to the low fertility of the transgenic mice, the experimental progress was slow, and it took nearly 10 years to complete the study to ensure the collection of the minimal required cell counts, as the inner ear contains very few cells. All efforts were made to reduce the number of animals used and to minimize their suffering.

### Methods details

#### Cell dissociation and sphere-forming culture

For each experiment, the OC were dissected from WT, *Bmi1*^±^, and *Bmi1*^*−/−*^ P2 neonatal mice. Each OC was carefully extracted from the cochlea with the anlage of the stria vascularis, the modiolus, and the tectorial membrane removed. All OCs were collected in phosphate-buffered saline (PBS; pH 7.4) on ice and incubated with 0.125% trypsin/EDTA (Invitrogen, Carlsbad, CA, USA) in PBS for 8 min at 37 °C. The enzymatic reaction was stopped by adding trypsin inhibitor (Serva, Heidelberg, Germany) and DNaseI solution (Invitrogen) in Dulbecco’s modified Eagle’s medium (DMEM)/high glucose and F12 media (mixed 1:1, DMEM/F12; Invitrogen). The cells were triturated carefully with plastic pipette tips (epTIPS Filter 20–300 μl; Eppendorf, Hamburg, Germany), and the cell suspension was passed through a 40-um cell strainer (BD Labware, San Jose, CA, USA).

For sphere culture, the cell suspensions were diluted to 20 cells/µl in Costar ultra-low attachment dishes (Coster, 3599) (Corning, NY, USA) with DMEM/F12 culture medium supplemented with N2 and B27 (Invitrogen) (Gibco, Thermo Fisher Scientific), EGF (20 ng/ml), bFGF (10 ng/ml, R&D Systems, Minneapolis, MN, USA), IGF-1 (50 ng/ml, R&D Systems), heparin sulfate (50 ng/ml, Sigma-Aldrich, St. Louis, MO, USA), and ampicillin (100 μg/mL, Sigma-Aldrich, St. Louis, MO, USA). Half of the culture medium was replaced every 48 h.

For the cell differentiation assay, the cell suspensions were cultured with a density of 50 cells/μl onto laminin-coated tissue culture plates (Greiner 35/10 mm four-well tissue culture dishes) using DMEM/F12 medium with N2, B27, and ampicillin without growth factors for an additional 7 days. EdU (10 μM, Invitrogen) was added during the culture to label the dividing cells. Immunofluorescence assays were conducted to analyze the types of differentiated cells 7 days after plating.

For the sphere differentiation assay, the spheres were cultured in sphere culture medium for 5 days and then transferred into laminin-coated tissue culture plates using DMEM/F12 medium with N2, B27, and ampicillin without growth factors for an additional 7 days. EdU (10 μM, Invitrogen) was added during the differentiation culture phase to label the dividing cells. Immunofluorescence assays were conducted to analyze the types of differentiated cells 7 days after plating.

### Western blot analysis

Total protein of the cochleae was isolated with RIPA (Beyotime) lysis buffer supplemented with PMSF (Beyotime). Protein concentrations were measured using a BCA protein assay kit (Beyotime), and proteins were separated on a BeyoGel™ Plus Precast PAGE Gel for Tris-Gly System (Beyotime) and transferred onto polyvinylidene difluoride membranes (Beyotime). The membranes were blocked with 10% nonfat dried milk in Tris-buffered saline with Tween 20 (TBST, 50 mM Tris–HCl (pH 7.4), 150 mM NaCl, and 0.1% Tween 20) for 1 h at room temperature and then blotted overnight with primary antibodies at 4 °C. The primary antibodies were as follows: Mouse anti-Cdkn2c/ p18^INK4C^ (1:1000 dilution) (Santa Cruz Biotechnology), Rabbit anti-GAPDH (HRP conjugated) (1:2000 dilution) (Abmart) (Supplementary Table 1).

### Genotyping and RT-qPCR

Total RNA was extracted from the cochlea with the anlage of the stria vascularis, the modiolus, and the tectorial membrane removed using TRIzol (Ambion). cDNA synthesis and qPCRs were performed with the PrimeScript™ II 1st Strand cDNA Synthesis Kit (Takara) and TB Green™ Premix Ex Taq™ II (Takara). Each PCR was carried out in triplicate, and the relative quantification of gene expression was performed using the ΔΔCT method with *Gapdh* as the endogenous reference. Primer pairs were designed using the online Primer3 software, and sequences are provided in Supplementary Table 1.

### Auditory brainstem response

Auditory brainstem response (ABR) thresholds were measured in anesthetized mice (100 mg/kg ketamine and 25 mg/kg xylazine sodium, i.p.) using a TDT System 3 (Tucker-Davis Technologies, Gainesville, FL, USA). Measurements were conducted in WT and *Bmi1*^*−/−*^ mice at P30, with thresholds assessed at four frequencies: 8, 16, 24, and 32 kHz.

### siRNA silencing

Spheres were transfected during the final stage of suspension sphere culture. Transfections were performed using 200 nM siRNA targeting *Cdkn2c* or a non-targeting control siRNA, both obtained from Sangon Biotech (Supplementary Table 1). The transfection was facilitated by Starvio siRNA transfection reagent (Starvio; Shanghai, China) and conducted in accordance with the manufacturer's protocol. The spheres were incubated with the transfection mixture for 24 h. To assess the efficiency of the siRNA transfection, quantitative real-time PCR (qRT-PCR) experiments were conducted 24 h after the completion of the transfection process.

### Immunofluorescence

Tissues/cells were fixed for 20 min at room temperature with 4% paraformaldehyde and then washed with PBS. Tissues/cells were blocked in blocking solution consisting of 10% donkey serum and 1%/0.1% Triton-X100 in PBS for 1 h. Incubation with primary antibodies that were diluted in blocking solution was performed overnight at 4 °C. The corresponding primary antibodies were as follows: rabbit anti- Myosin VIIa (1:500 dilution; Proteus Biosciences, Ramona, CA, USA), goat anti-Sox2 (1:500 dilution, Santa Cruz Biotechnology, Dallas, TX, USA), mouse anti-p18^INK4C^ (1:500 dilution; Santa Cruz Biotechnology, Dallas, TX, USA), mouse anti-Histone H3 (tri methyl K27) (1:500 dilution; Abcam, Cambridge, UK) and anti-Caspase3 (1:500 dilution; Abcam, Cambridge, UK). The following day, tissues /cells were rinsed with PBS and then incubated with Alexa Fluor 488–, 594–, and/or 647–conjugated secondary antibodies (Jackson Immuno Research, West Grove, PA, USA) at 1:500 dilution for 1 h at room temperature. Nuclei were labeled with 4, 6-diamidino-2-phenylindole (DAPI; 1:1000 dilution; Invitrogen, Carlsbad, CA, USA) for 20 min at room temperature (Supplementary Table 1). After washing with PBS, tissues/cells were mounted in antifade fluorescence mounting medium and cover slipped. Specimens were examined by confocal fluorescence microscopy (Leica SP8, Heidelberg, Germany).

### RNA-sequencing and analysis

Total RNA was extracted from six OCs per sample using the AllPrep DNA/RNA/Protein Mini kit (QIAGEN). Three independent biological replicates were performed for each genotype (WT and *Bmi1*^−/−^ P2 neonatal mice). The double-strand cDNA was synthesized from the total RNA LT Sample Prep Kit v2 (Illumina). Illumina adapters were ligated to the cDNA molecules after end repair, and the ligated cDNA was cleaned up with AmpureBeads (Beckman). The library was amplified using 10 cycles of PCR for the enrichment of adapter-ligated fragments, and transcriptome sequencing was carried out with an Illumina-Hiseq2500 system (Illumina).

Preliminary processing of raw reads was performed using Casava 1.8 (Illumina), and adapters and low-quality bases were trimmed using trimmomatic (version 0.23). Trimmed reads for each sample were aligned to the *Mus musculus* UCSC mm10 genome using Hisat2, and aligned reads were counted with HTseq [24] according to UCSC mm10 annotation. Differential expression analysis between conditions was performed using the DESeq2 R package [25], and genes with an adjusted p-value (padj) < 0.05 and fold change > 1.5 were considered to be differentially expressed. GO analysis of significantly differentially expressed genes was performed with DAVID (http://david.abcc. ncifcrf.gov/home.jsp). The Pheatmap R package was used to generate heatmaps, and protein–protein interaction network analysis of differentially expressed genes in the Hippo, Notch, and Wnt signaling pathways was performed using STRING software (Figure S3).

### ATAC-sequencing and analysis

OCs from WT and *Bmi1*^*−/−*^ P2 neonatal mice were dissected separately. Ten mice (20 cochleae) from each group were pooled and then subjected to sequencing. Nuclei isolation was performed using lysis buffer containing 10 mM Tris–HCl (pH 7.4), 10 mM NaCl, 3 mM MgCl2, and 0.1% IGEPAL CA-630. The ATAC-seq library preparation was conducted using the Hyperactive ATAC-Seq Library Prep Kit for Illumina (Vazyme, TD711) following the manufacturer's protocol. Each sample consisted of 2 × 10^4^ cells.

Cells were washed with 500 μl PBS and centrifuged at 500 × g for 5 min at room temperature. The cell pellets were then resuspended in 50 μl of cold lysis buffer and incubated on ice for 10 min to isolate the nuclei. After centrifugation at 500 × g at 4 °C for 5 min, the nuclei were subjected to a transposition reaction by incubating with 50 μl Tn5 transposome/Transposition reaction mix at 37 °C for 30 min. During this step, Tn5 transposase simultaneously fragmented the DNA and inserted adapters into open chromatin regions.

The tagmented DNA was purified using VAHTS DNA Clean Beads to remove proteins and other impurities. The purified DNA underwent two washes with 200 μl of fresh 80% ethanol and was eluted in 26 μl of nuclease-free water. PCR amplification was performed to enrich the adapter-ligated DNA fragments. The amplification protocol consisted of 72 °C for 3 min; 95 °C for 3 min; 14 cycles of 98 °C for 10 s, 60 °C for 5 s, and 72 °C for 1 min; followed by a hold at 12 °C. The amplified library was further purified using VAHTS DNA Clean Beads, washed twice with 80% ethanol, and eluted in 22 μl of nuclease-free water.

The ATAC-seq libraries were sequenced on an Illumina NovaSeq 6000 platform with paired-end 150 bp reads. Low-quality bases and adapter sequences were removed, and the data were quality-controlled using FastQC software. The quality-controlled reads were mapped to the reference genome using BWA-MEM software. Peak calling was performed with MACS2. To account for differences in sequencing depth and library complexity between samples, read counts were normalized. Specifically, we used the bamCoverage function from the Deeptools suite to generate normalized bigWig files from the alignment (BAM) files, using reads per genomic content (RPGC) normalization. The peaks were annotated by associating them with their nearest gene elements using HOMER software. Functional enrichment analysis of the peak-associated genes was then conducted using databases such as Gene Ontology (GO) and KEGG (Figure S3).

### ChIP-Sequencing and analysis

Twenty OCs from WT and *Bmi1*^−/−^ P2 neonatal mice were separately pooled and sonicated to shear crosslinked chromatin. The chromatin extract was then incubated with anti-H3K27me3 (Abcam) overnight at 4 °C followed by the addition of Protein A/G Dynabeads (1:1, Invitrogen). Immunoprecipitated complex was eluted from the beads and de-crosslinked, and chromatin was purified using the MinElute PCR Purification Kit (Qiagen). Purified DNA was used to prepare ChIP-seq DNA libraries for Illumina sequencing.

Raw ChIP-seq reads were trimmed using the trimmomatic tool [26] and then aligned to the *Mus musculus* UCSC mm10 genome using Bowtie2. Duplicated reads were removed, and only uniquely mapping reads were retained for further analysis. The MACS2 (version 2.1.1) callpeak algorithm was used to identify regions of ChIP-seq enrichment over background. To ensure comparability between samples, we normalized for sequencing depth. Normalized signal tracks (bigWig files) were generated from the alignment files using the bamCoverage function in Deeptools with reads per genomic content (RPGC) normalization. Only differential binding peaks with fold change ≥ 1.5 and FDR < 0.05 were used for further study. A correlation heatmap based on the affinity scores for ChIP-seq samples was also plotted using the Pheatmap R package with the default settings.

Deeptools was used to normalize all genes of the two sets of data according to the gene length and to calculate the average signal values of all genes at each site within the range of 3 kb upstream and 3 kb downstream of the transcription start site (TSS). DEGseq was used to analyze the differences in peaks between the two groups of data, and Deeptools was used to calculate the signal values of all peaks at each site in the normalized difference peak region, and a heat map was drawn (Figure S2).

### Multiomic data processing

We conducted differential analysis on RNA-seq, ATAC-seq, and ChIP-seq datasets using edgeR in R. Features were considered significant if they met the criteria of |log2FC|≥ 0.25 and p-value < 0.01. For the key differentially expressed genes identified from RNA-seq analysis, we performed transcription factor enrichment analysis using TRUST. The results from these analyses were then integrated to construct a regulatory network. We utilized Cytoscape for network visualization, where genes and transcription factors were represented as nodes, and regulatory relationships as edges.

The transcriptomic sequencing data, chromatin immunoprecipitation (ChIP) sequencing data, and assay for transposase-accessible chromatin (ATAC) sequencing results have been deposited in the Gene Expression Omnibus (GEO) database. These datasets are accessible under the following GEO accession numbers: GSE279539 (ChIP sequencing), GSE279541 (transcriptomic sequencing), and GSE279758 (ATAC sequencing).

### Quantification and statistical analysis

All data were statistically analyzed using GraphPad Prism. The significance of the differences between two groups was calculated by unpaired Student’s* t*-test (two-tailed). *P* < 0.05 and *P* < 0.01 were considered statistically significant and highly significant, respectively. All data are presented as the mean ± S.E.M.

## Results

### Impaired sphere-forming capability in ***Bmi1***^−/−^ sensory epithelial cells

**Fig. 1:**
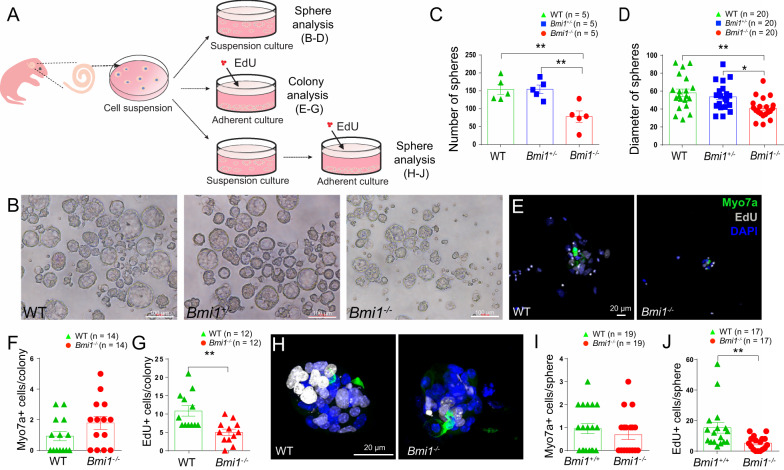
Bmi1 deficiency impairs sphere-forming capability in the organ of Corti (OC). **A** Experimental procedure for sphere formation and differentiation culture. **B** Representative images of spheres derived from dissociated P2 neonatal WT, *Bmi1*^±^, and *Bmi1*^*−/−*^ OC after 5 days of suspension culture. Scale bar = 100 μm. **C**, **D** Fewer and smaller spheres were observed in *Bmi1*^−/−^ OC compared to WT and *Bmi1*^±^ group. **E** Immunofluorescence staining of differentiated and proliferating cells derived from WT and *Bmi1*^−/−^ colonies. **F** The number of Myosin VIIa + cells (hair cells) showed no significant difference in WT and *Bmi1*^−/−^ colonies. **G** The number of EdU + cells (proliferating cells) were significantly decreased in *Bmi1*^−/−^ colonies when compared with WT. **H** Immunofluorescence staining of differentiated and proliferating cells generated from WT and *Bmi1*^−/−^ spheres. **I** The number of Myosin VIIa + cells showed no significant difference in WT and *Bmi1*^−/−^spheres. **J** The number of EdU + cells were significantly decreased in *Bmi1*^−/−^ spheres when compared with WT. Myosin VIIa labels hair cells in green. EdU labels proliferating cells in gray. DAPI labels cell nuclei in blue. Data are presented as Mean ± S.E.M. *p < 0.05, **p < 0.01. "n" represents the number of biological replicates analyzed

Previous studies have demonstrated that the auditory sensory epithelium of the inner ear harbors progenitor cells capable of forming spheres with stem cell-like properties [27–30]. To characterize the role of *Bmi1* in this context, we utilized postnatal *Bmi1*^−/−^ transgenic mice and wild-type (WT) mice. Cell suspensions from the OC explants were cultured in vitro (Fig. 1A). Consistent to our previous findings [22], Our results (Fig. 1B–D) reveal a significant reduction in both the number and size of spheres formed from *Bmi1*^*−/−*^ progenitors compared to WT controls and *Bmi1*^±^ *group*, indicating that *Bmi1* deficiency hampers the proliferation of sensory epithelial cells in vitro. Specifically, the average sphere counts were 154 ± 31.2 for WT (n = 5), 154 ± 26.2 for *Bmi1*^±^ mice (n = 5), and 78 ± 35.9 for *Bmi1*^*−/−*^ mice (n = 5) with each group starting from 20,000 cells respectively. Furthermore, sphere diameters averaged 58 ± 4.2 µm in WT (n = 20), 53 ± 3.4 µm in *Bmi1*^±^ mice (n = 20), and 40 ± 2.6 µm in *Bmi1*^−/−^ (n = 20), highlighting a marked reduction in sphere size due to *Bmi1* deficiency.

To investigate whether *Bmi1*-deficient auditory sensory epithelial cells could differentiate into hair cells, we cultured cell suspension from the OC at a density of 50 cells /μl on laminin-coated plates under adherent conditions, adding EdU to label proliferating cells (Fig. 1A). After 7 days, the cells were immunostained with Myosin VIIa and Sox2, markers for hair cell and supporting cell, respectively. Our analysis indicated no significant difference in the number of Myosin VIIa + cells between WT and *Bmi1*^−/−^ colonies (0.9 ± 0.3 (n = 14) in WT and 1.8 ± 0.4 (n = 14) in *Bmi1*^−/−^); however, *Bmi1*^−/−^ colonies exhibited a significant decrease in proliferating EdU + cells compared to WT (10 ± 1.4 EdU + cells per WT sphere (n = 12) vs. 5 ± 0.8 EdU + cells per *Bmi1*^−/−^ sphere (n = 12), **p < 0.01, Figs. 1E–G).

Further investigations were conducted by transferring spheres cultured for 5 days in non-adherent conditions to laminin-coated 4-well dishes in serum-free adherent conditions for an additional 7 days, with continued EdU exposure (Fig. 1A). Post-cultured, cells were immunostained with Myosin VIIa. While the number of Myosin VIIa + cells was comparable between the two groups (the number of Myosin VIIa + cells per colony in WT and *Bmi1*^*−/−*^ spheres were 0.9 ± 0.2 (n = 19) and 0.68 ± 0.2 (n = 19); Fig. 1H–I), *Bmi1*^−/−^ spheres showed a significantly reduced number of EdU + proliferating cells compared to WT (15 ± 3.5 EdU + cells per WT sphere (n = 17) vs. 5 ± 1.0 EdU + cells per *Bmi1*^*−/−*^ sphere (n = 17), **p < 0.01, Fig. 1J).

In our preliminary results, we found that the inner ear's OC in newborn Bmi1 knockout mice contains both hair cells and supporting cells [22], which is consistent with the results from the sphere-forming experiments in vitro. These findings indicate that *Bmi1* deficiency does not affect the differentiation of hair cells from progenitors, it significantly reduces the proliferative capability of sensory epithelial cells in the inner ear, which could be one of the reasons for the hearing loss observed in Bmi1 knockout mice and the apoptosis of hair cells in adult mice (Figure S1). This prompt further investigation into the role of Bmi1, particularly its involvement in histone modification, such as the characteristics of H3K27me3 [12, 31, 32] and gene expression regulation within the inner ear.

### Gene expression profile in cochleae of *Bmi1*^−/−^ mice

**Fig. 2:**
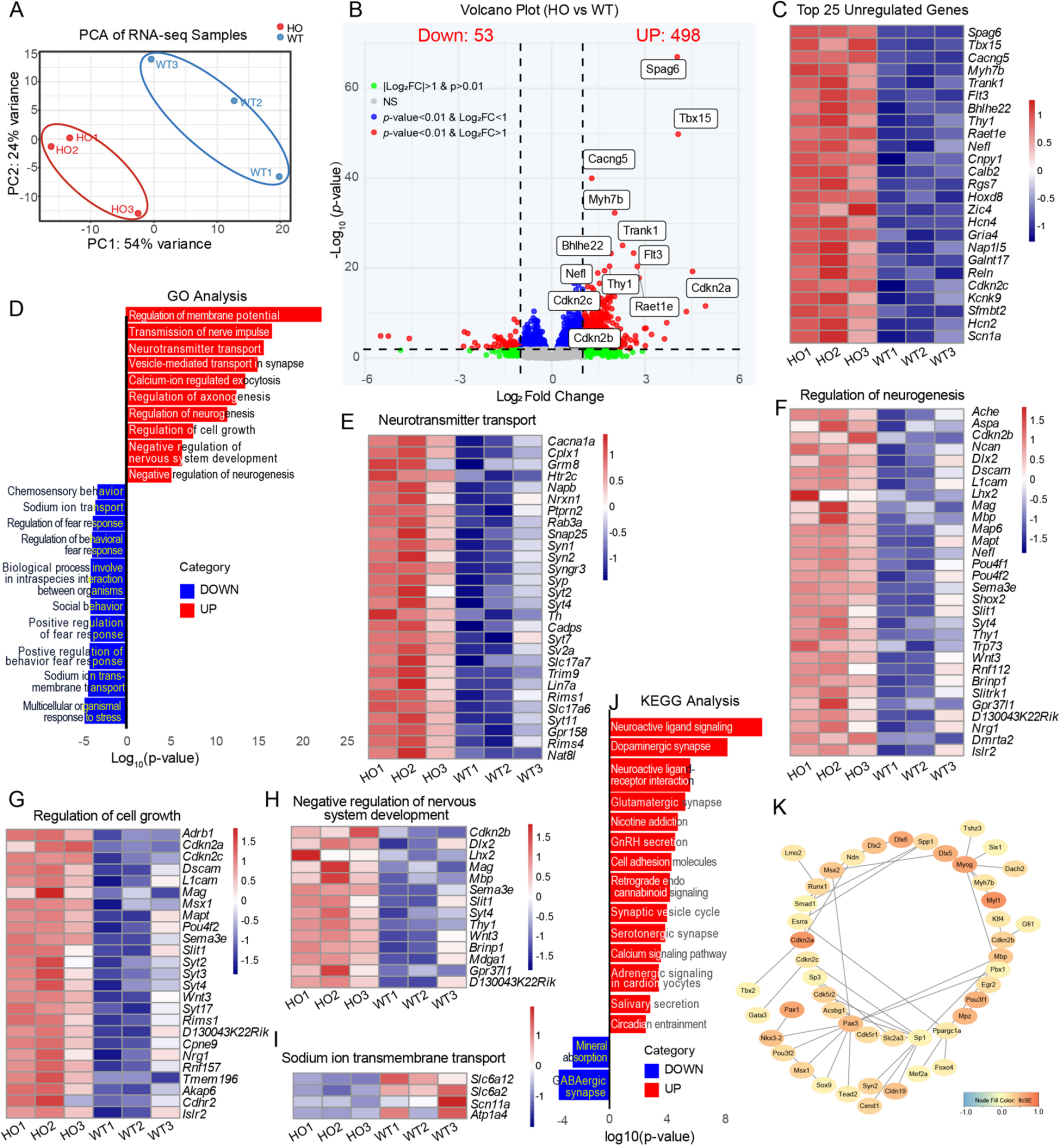
Changes in gene expression in the sensory epithelium cells of Bmi1 knockout mice. **A** Principal component analysis. **B** Volcano plot of the differentially expression genes in *Bmi1*^*−/−*^ mice compared to WT mice. **C** Heatmap of the top 25 most significantly differentially expressed genes. **D** Top 10 GO terms for upregulated genes and 10 GO terms for downregulated genes in *Bmi1*^*−/−*^ mice compared to WT mice. **E**–**H** Heatmap showing genes that were significantly upregulated in the GO terms of neurotransmitter transport, regulation of neuroscience, regulation of cell growth, and negative regulation of nervous system development in *Bmi1*^*−/−*^ mice compared to WT mice. **I** Heatmap showing genes that were significantly decreased in the GO terms of regulation of sodium ion transmembrane transport in *Bmi1*^*−/−*^ mice compared to WT mice. **J** KEGG pathway analysis of differential expressed genes between *Bmi1*^*−/−*^ and WT mice. **K** A representative gene interaction network diagram showed an increased expression of *Cdkn2c* in *Bmi1*^−/−^ mice compared to WT mice, with Cdkn2c interacting with other proteins. In the Figure, "HO" represented *Bmi1*^*−/−*^group

To investigate the impacts of Bmi1 knockout on gene expression, RNA sequencing was conducted on the OC from both WT and *Bmi1*^*−/−*^ P2 neonatal mice. Principal component analysis (PCA) of the RNA-seq data revealed that the first principal component (PC1/Dim1) explained 54% of the total variance between the WT and *Bmi1*^*−/−*^ cochleae, illustrating distinct genomic responses to *Bmi1* deficiency (Fig. 2A). In the comparative analysis to WT mice, the *Bmi1*^*−/−*^ group exhibited elevated expression level in 498 genes and decreased expression level in 53 genes (Fig. 2B). This indicated that the changes in gene expression caused by *Bmi1* gene knockout were mainly characterized by upregulation of expression. Figure 2C displayed the heatmap of the top 25 most significantly differentially expressed genes, showing distinct expression patterns between WT and *Bmi1*^*−/−*^ groups. The top 25 upregulated genes in Bmi1 KO versus WT groups—including *Spag6, Tbx15, Cdkn2c*, and *Reln*—are functionally implicated in cell-cycle regulation, neural development, ion transport, and epigenetic modulation (Supplementary Table S2). GO analysis of these up-regulated genes underscored their roles in processes such as neurotransmitter transport, regulation of neurogenesis, regulation of cell growth and negative regulation of nervous system development (Fig. 2D–G). GO analysis of down-regulated genes underscored their roles in processes such as multicellular organismal response and sodium ion transmembrane transport (Fig. 2D and H). Furthermore, KEGG pathway analysis highlighted enrichment in pathways related to the neuroactive ligand signaling, neuroactive ligand-receptor interaction (Fig. 2J). Gene interaction network analysis revealed that the expression of *Cdkn2c* was increased in *Bmi1*^*−/−*^ mice compared to WT mice and *Cdkn2c* was interacted with other proteins (Fig. 2K).

### Identification and characterization of H3K27me3 signals in the auditory sensory epithelium of neonatal mice

**Fig. 3:**
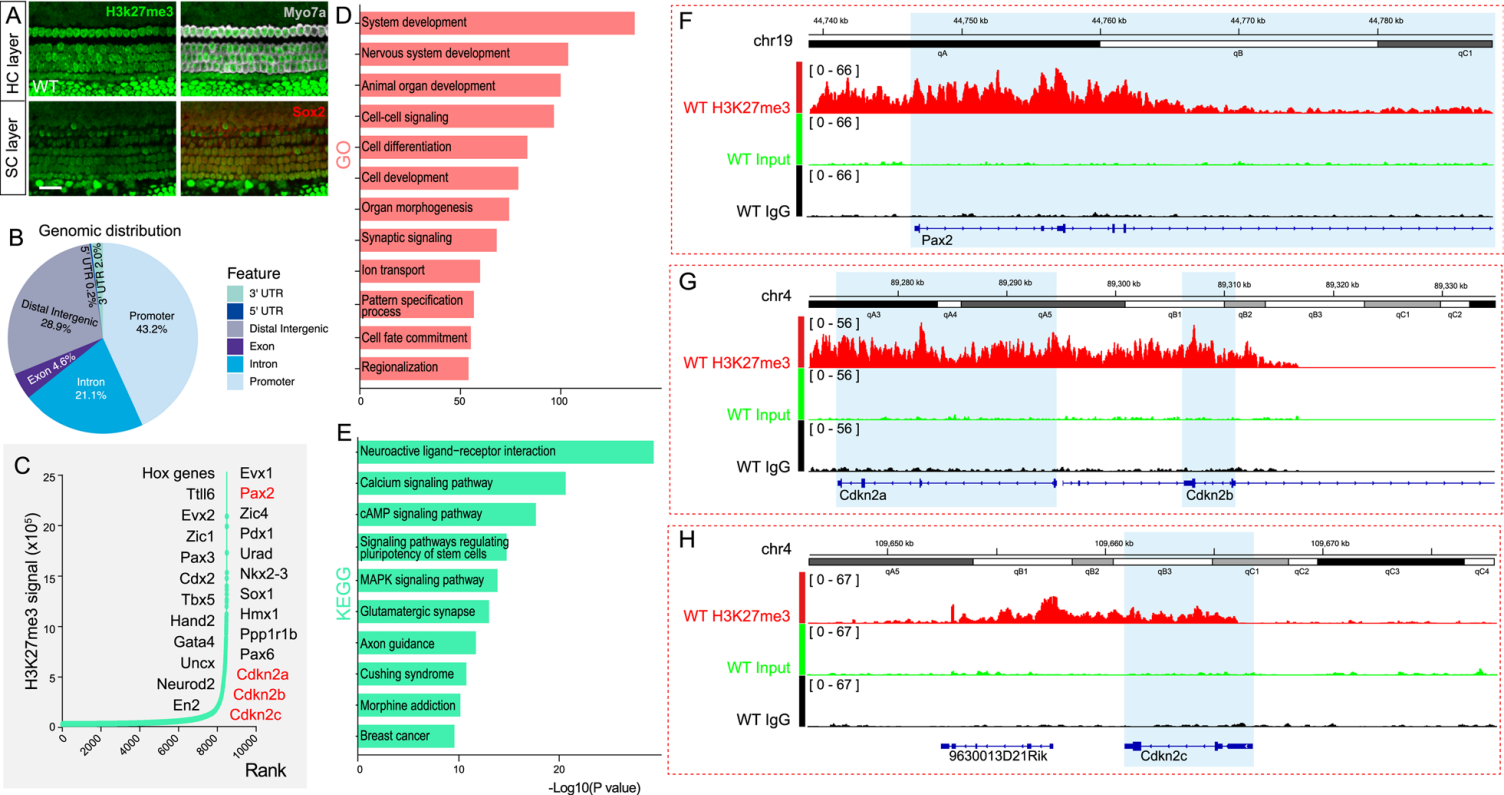
H3K27me3 involvement in the regulation of gene transcription in the inner ear. **A** H3K27me3 was ubiquitously expressed in the auditory sensory epithelium of P2 neonatal mice inner ear. Hair cells are labeled with Myosin VIIa in gray, H3K27me3 is labeled in green, and support cells are labeled with Sox2 in red. **B** Genomic distribution of H3K27me3 signals in the auditory sensory epithelium of P2 neonatal mice inner ear. **C** Representation of H3K27me3-rich genes ranked by Mean Reciprocal Rank. Genes highlighted in red (*Pax2*, *Cdkn2a*, *Cdkn2b*, and *Cdkn2c*) were specifically labeled because of their well-established functions in regulating cell proliferation and the cell cycle. **D** GO analysis of genes with H3K27me3 signals. **E** KEGG pathway analysis of genes with H3K27me3 signals. Screenshot showing the H3K27me3 signals of the *Pax2* (**F**), *Cdkn2a*, *Cdkn2b* (**G**) and *Cdkn2c* (**H**) gene sequences and nearby regions

We initiated our study by conducting immunofluorescence staining to characterize the presence of H3K27me3 protein in the cochlea of P2 neonatal mice. Our results indicated a ubiquitous expression of H3K27me3 across both hair cells and supporting cells in the auditory sensory epithelium, suggesting a general establishment of H3K27me3 modification in these regions of neonatal mice (Fig. 3A). To further investigate the specific genomic patterns of H3K27me3 modification, we performed ChIP sequencing on inner ear tissue from WT neonatal mice. Figure 3B illustrates the genomic distribution of H3K27me3 peaks in the auditory sensory epithelium of P2 neonatal mice inner ear, which are enriched in promoter regions.

Our next step was to identify and rank regions with high levels of H3K27me3 modification, referred to as methylation-rich regions (MRRs). Following the method described by Cai et al. [33], we clustered nearby H3K27me3 peaks and ranked these clusters based on their average signal intensity. The analysis identified the top 25 MRRs, which overlapped with key regulatory genes including *Pax2, Cdkn2a*, *Cdkn2b*, and *Cdkn2c*, all known for their roles in cell proliferation and cell cycle regulation (Fig. 3C).

GO analysis of the genes associated with enriched H3K27me3 signals highlighted their involvement in critical biological processes, such as system development, nervous system development, organ development, and cell–cell signaling (Fig. 3D). KEGG pathway analysis highlighted enrichment in pathways related to signaling pathways regulating pluripotency of stem cells (Fig. 3E). To further substantiate the presence of enriched H3K27me3, we examined four representative MRRs overlapping with the genes *Pax2*, *Cdkn2a, Cdkn2b and Cdkn2c*. *Pax2*, a crucial transcription factor in organogenesis including the inner ear, showed significant H3K27me3 signals upstream of *Pax2* TSS (Fig. 3F). Conversely, *Cdkn2a*, *Cdkn2b* and *Cdkn2c*, all known as important tumor suppressor genes, displayed widespread H3K27me3 signals throughout their genomic sequence (Fig. 3G, H). These findings illustrate the extensive involvement of H3K27me3 in regulating genes critical for inner ear development and cellular functions, providing insights into the epigenetic control mechanisms within the auditory sensory epithelium of neonatal mice.

### Increased chromatin accessibility profiling by ATAC-seq and reduced genome-wide H3K27me3 signals by ChIP-seq in *Bmi1*^*−*/−^ mice In vivo

The data described above suggest that the knockout of the *Bmi1* gene caused changes in the transcription levels of a series of genes in inner ear sensory epithelial cells, with a predominant upregulation of gene expression. Bmi1 serves as a key regulatory component of the PRC1 complex, which is required to maintain the transcriptionally repressed state of many genes throughout development via chromatin remodeling and histone modification[34]. The assay of transposase-accessible chromatin using sequencing (ATAC-seq) provides a way to detect the unique chromatin landscape [35], enabling the study of the effects of *Bmi1* gene knockout on the regulation of inner ear sensory epithelial cells at the chromatin level. ATAC-seq data was extracted from OC of WT and *Bmi1*^*−/−*^ P2 neonatal mice. Considering the crucial roles that Bmi1 and H3K27me3 play in the development and differentiation of the inner ear, we sought to explore their potential interrelationship further. Using ChIP-seq, we compared H3K27me3 signals in the OC from neonatal *Bmi1*^*−/−*^ mice to those from WT neonatal mice.

**Fig. 4:**
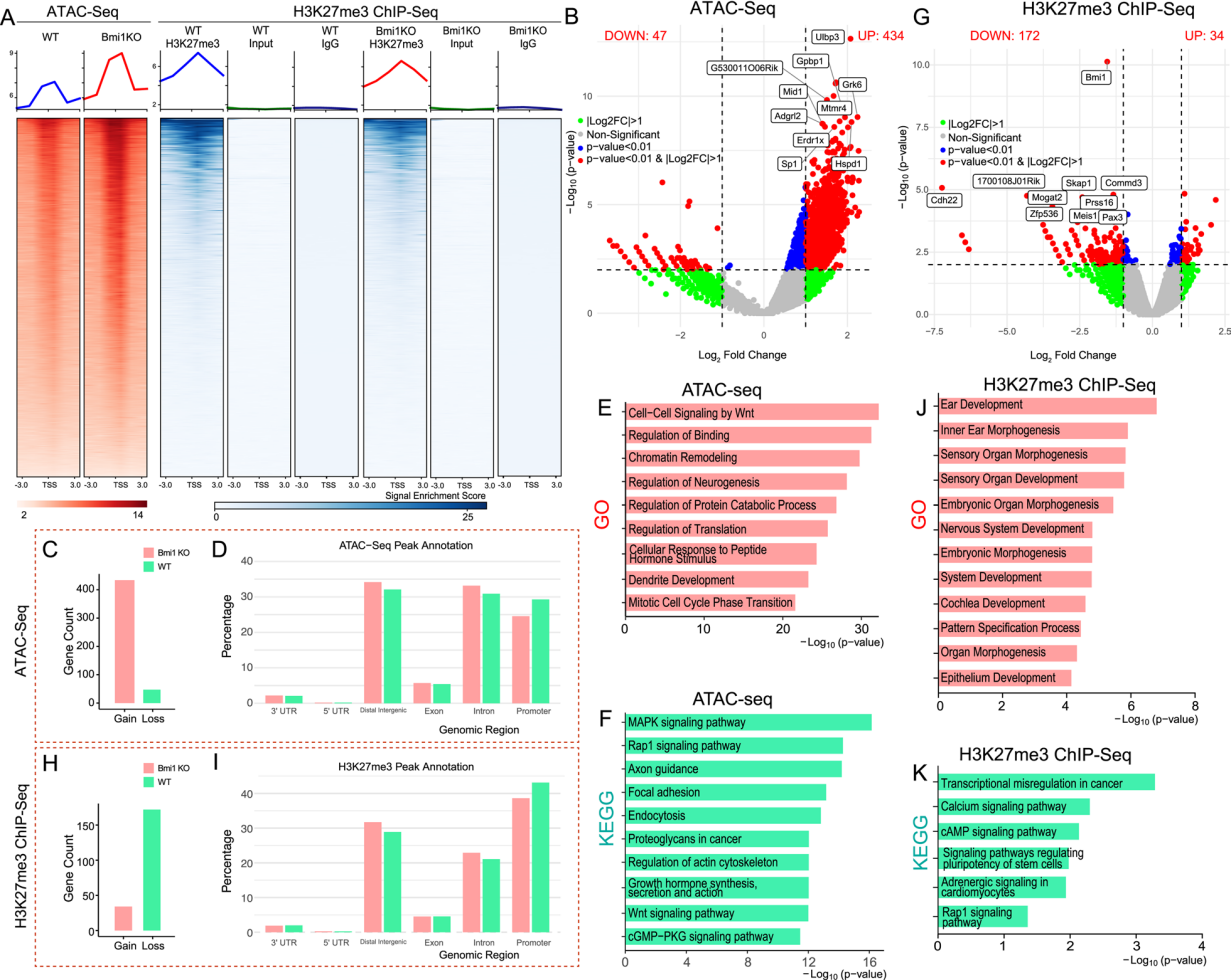
Bmi1 knockout affected chromatin accessibility and the H3K27me3 marks in inner ear sensory hair cells. **A** Heatmaps showing the distribution of ATAC signals and H3K27me3 marks near transcription start sites (TSS) in WT and *Bmi1*^*−/−*^ group. The distribution of ATAC signal density was much higher in *Bmi1*^*−/−*^ group when compared with WT. The distribution of H3K27me3 signals density was lower in *Bmi1*^*−/−*^ group when compared with WT. **B** Volcano plot of genes identified with differential chromatin accessibility between WT and *Bmi1*^*−/−*^ group. Green dots: |Log₂FC|> 1, Gray dots: Not significant (NS), Blue dots: p-value < 0.01, Red dots: p-value < 0.01 and |Log₂FC|> 1. **C** Numbers of genes with differential chromatin accessibility observed in *Bmi1*^*−/−*^ group when compared with WT group. **D** Genomic distribution of all ATAC-seq peaks in WT and *Bmi1*^*−/−*^ cochleae. **E** Bar plot illustrating the GO terms enriched in genes with increased chromatin accessibility regions in the *Bmi1*^*−/−*^ group compared to the WT group. **F** Bar plot illustrating the KEGG terms enriched in genes with increased accessibility regions in the *Bmi1*^*−/−*^ group compared to the WT group. **G** Volcano plot of genes identified with differential H3K27me3 marks between WT and *Bmi1*^*−/−*^ group. Green dots: |Log₂FC|> 1, Gray dots: Not significant (NS), Blue dots: p-value < 0.01, Red dots: p-value < 0.01 and |Log₂FC|> 1. **H** Numbers of with differential H3K27me3 marks observed in *Bmi1*^*−/−*^ group when compared with WT group. **I** Genomic distribution of H3K27me3 ChIP-seq peaks between WT and *Bmi1*^*−/−*^ group. **J** Bar plot illustrating the GO terms enriched in genes with decreased H3K27me3 marks in the *Bmi1*^*−/−*^ group compared to the WT group. **K** Bar plot illustrating the KEGG terms enriched in genes with decreased H3K27me3 marks in the *Bmi1*^*−/−*^ group compared to the WT group

The heatmap in Fig. 4A shows the average distribution of reads across a ± 3 kb region of the transcription start site (TSS) for all genes in WT and *Bmi1*^*−/−*^ sensory epithelial cells, as calculated and normalized by Deeptools. The results indicated that ATAC signals mainly concentrated in the TSS proximal region, and signals were more abundant in the *Bmi1*^−/−^ group. However, we noted a significant reduction in the intensity of H3K27me3 signals within the central region of the peaks in the *Bmi1*^*−/−*^ group compared to the WT control (Fig. 4A). Differential analysis of the signals revealed that 434 genes exhibited increased ATAC signals in *Bmi1*^−/−^ group when compared with WT group, while the number of genes with decreased signals was very low (Fig. 4B, C). Figure 4D showed the percentage of ATAC-seq peaks annotated to different genomic regions (including promoters, introns, exons, distal intergenic regions) in each group. GO analysis of these differential genes suggested that their functions might be related to cell–cell signaling by Wnt, regulation of binding, chromatin remodeling and mitotic cell cycle phase transition (Fig. 4E). KEGG pathway analysis indicated that these genes are involved in MAPK signaling pathway, Rap1 signaling pathway and Axon duidance (Fig. 4F). Differential peak analysis of ChIP-seq signals was performed using DEGseq to discern variations between the WT and *Bmi1*^−/−^ group, showing that 172 peak related genes in the *Bmi1*^*−/−*^ group exhibited a loss of H3K27me3 signals, whereas 34 peaks showed an increase (Fig. 4G, H). Figure 4I showed the percentage of H3K27me3 peaks annotated to different genomic regions (including promoters, introns, exons, distal intergenic regions) in each group. GO analysis of the genes with decreased H3K27me3 marks indicated their association with crucial biological processes such as ear development, inner ear morphogenesis, and embryonic organ morphogenesis (Fig. 4J). Further, KEGG pathway analysis indicated that these genes are involved in numerous key signaling pathways, including transcriptional mis-regulation in cancer, the calcium signaling pathway, and the pathways regulating pluripotency of stem cells (Fig. 4K). These findings underscore that Bmi1 knockout significantly affects the chromatin accessibility and the distribution of H3K27me3 marks across the genome, with potential consequences on the regulation of gene expression critical for the proliferation and development of cochlear sensory epithelial cells. Such alterations in the epigenetic landscape suggest a disrupted regulatory mechanism in *Bmi1*^−/−^ mice that could affect normal sensory functions and development in the inner ear.

### Multiple omics analysis showed that *Cdkn2c* was involved in the fate regulation of sensory epithelial cells of neonatal mice cochleae.

**Fig. 5:**
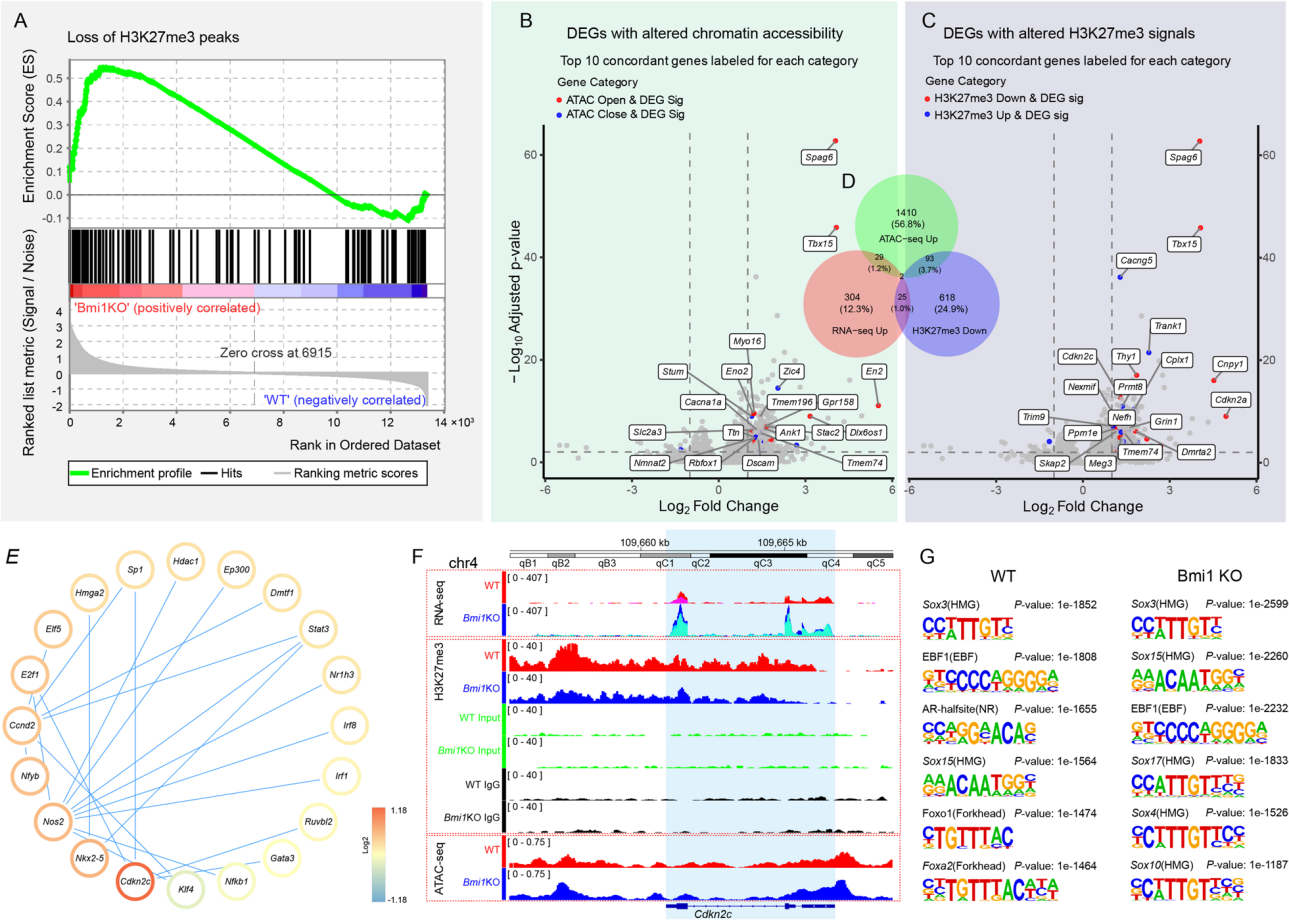
Integrated analysis of RNA-seq, ChIP-seq and ATAC-seq. **A** Gene Set Enrichment Analysis of genes with decreased H3K27me3 marks in *Bmi1*^*−/−*^ group. **B** Volcano plots indicating the overlap of gene identities from differential genes and peak annotations from RNA-seq and ATAC-seq in the *Bmi1*^*−/−*^ group compared to the WT group, respectively. Gene and peak annotations were categorized as either increased or decreased based on the direction of expression and accessibility change. **C** Volcano plots indicating the overlap of gene identities from differential genes and peak annotations from RNA-seq and H3K27me3-ChIP-seq in the *Bmi1*^*−/−*^ group compared to the WT group, respectively. Gene and peak annotations were categorized as either increased or decreased based on the direction of expression and H3K27me3 signals. **D** Analysis of the overlap between upregulated genes, loss of H3K27me3, and increased chromatin accessibility in *Bmi1*^*−/−*^ group. **E** Network analysis of the interaction between genes with increased expression in RNA-seq and genes with decreased H3K27me3 marks in ChIP-seq in the *Bmi1*^*−/−*^ group compared to the WT group. **F** Screenshot showing the RNA expression peak, chromatin accessibility, and H3K27me3 signals of the *Cdkn2c* gene in the genome of WT and *Bmi1*^*−/−*^groups. **G** Top six motifs from ATAC-seq data in the WT group and the *Bmi1*^*−/−*^ group, respectively

Given the well-established role of H3K27me3 modification in gene silencing [36], we hypothesized that the observed reduction in H3K27me3 signals in *Bmi1*^−/−^ mice would lead to increased chromatin accessibility and transcriptional activity. Correlating the 172 genes with decreased H3K27me3 signals to the 617 genes with increased expression, Gene Set Enrichment Analysis (GSEA) identified a significant correlation between the upregulation of gene expression and the loss of H3K27me3 peaks following Bmi1 knockout (Fig. 5A).

Integrated RNA-seq and ATAC-seq analysis revealed that genes with upregulated mRNA expression in the *Bmi1*^−/−^ group were predominantly associated with increased chromatin accessibility (Fig. 5B). Integrated RNA-seq and ChIP-seq analysis showed that, compared to the WT group, genes with upregulated mRNA expression were mainly distributed on the side with reduced H3K27me3 signals in the *Bmi1*^*−/−*^ group (Fig. 5C). Furthermore, we performed an integrated analysis to identify genes that were upregulated in conjunction with a decrease in H3K27me3 signal and an increase in ATAC-seq signal in *Bmi1*^*−/−*^ group. 29 genes were identified with upregulated mRNA expression accompanied by an increase in ATAC-seq signal, 93 genes were identified with upregulated mRNA expression along with a decrease in H3K27me3 signal, and 25 genes were identified with increased ATAC-seq signal concurrent with a reduction in H3K27me3 signal. 2 genes were identified with upregulated mRNA expression along with a decrease in H3K27me3 signal and increase in ATAC-seq signal (Fig. 5D). Compared to the WT group, interaction network analysis of genes with upregulated mRNA levels and genes with decreased H3K27me3 signals in the Bmi1 knockout group revealed that *Cdkn2c*, a cell cycle inhibitory regulator, was significantly upregulated at the mRNA level in the *Bmi1*^*−/−*^ group. Additionally, *Cdkn2c* was found to interact with *E2f1*, *Sp1*, and *Ruvbl2* (Fig. 5E). *Cdkn2c* in the *Bmi1*^−/−^ mice showed a markedly increased expression peaks compared to controls (Fig. 5F). This was accompanied by a decrease in H3K27me3 signals and slightly increase in chromatin accessibility across the entire gene length and extended 10 kb upstream (Fig. 5F), suggesting that the reduction of H3K27me3 signals due to Bmi1 knockout potentially facilitates the transcriptional activation of *Cdkn2c*. Transcription factor binding sites were identified from RNA-seq data from WT group and *Bmi1*^*−/−*^ group respectively. The results showed that the top six transcription factor binding motifs in the WT group were *Sox3*, *EBF1*, *AR-halfsite*, *Sox15*, *Foxo1*, and *Foxa2*. In contrast, the top six transcription factor binding motifs in the *Bmi1*^*−/−*^ group were *Sox3*, *Sox15*, *EBF1*, *Sox17*, *Sox4*, and *Sox10*. Bmi1 knockout may affect the activity of transcription factors binding to these motifs, thereby influencing the expression of downstream genes (Fig. 5G). These results indicate a significant impact of Bmi1 knockout on the epigenetic landscape and gene expression profiles in neonatal mouse cochleae, particularly highlighting the role of H3K27me3 in regulating genes essential for neural development and function through regulating the chromatin accessibility.

### Inhibition of *Cdkn2c* enhances proliferation in epithelial cells of *Bmi1*^*−/−*^ cochleae

**Fig. 6:**
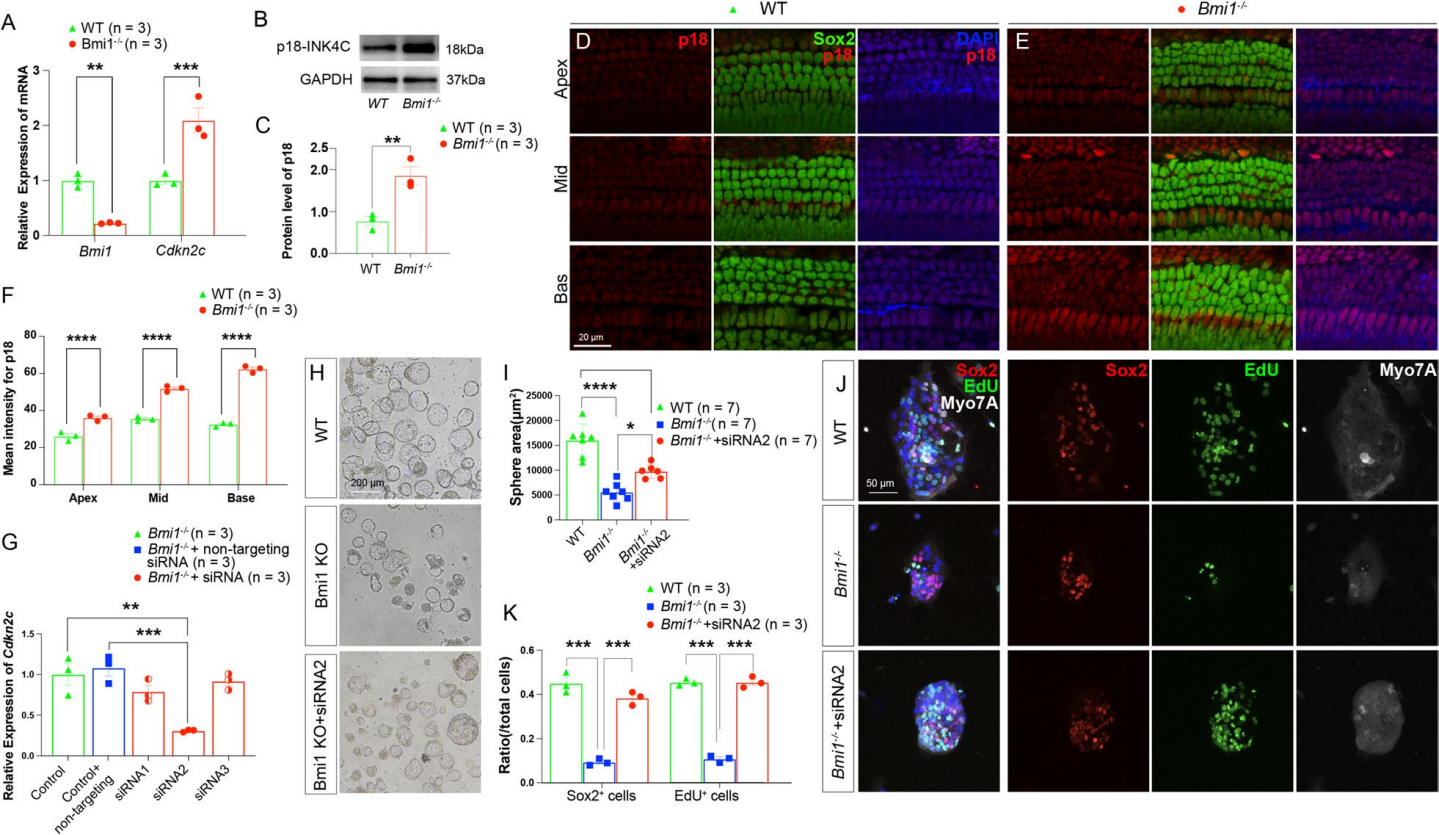
Inhibition of *Cdkn2c* promoted the proliferating ability in epithelial cells of *Bmi1*^*−/−*^ OC. **A** RT-qPCR showed that the mRNA expression levels of *Bmi1* was significantly decreased and the mRNA expression levels of *Cdkn2c* was significantly increased in *Bmi1*^*−/−*^ group when compared with WT group. **B** Western blots showed that the protein expression level of p18^INK4c^ was significantly increased in *Bmi1*^*−/−*^ group when compared with WT group. **C** Relative quantification of p18^INK4c^ density in WT and *Bmi1*^*−/−*^ group based on Western Blots. **D**, **E** Immunofluorescence staining showing increased p18^INK4c^ expression in supporting cells in *Bmi1*^*−/−*^ group. Apex: apical turn; Mid: middle turn; Base: basal turn. **F** Relative immunofluorescence quantification of p18^INK4c^ in WT and *Bmi1*^*−/−*^ group. **G** Relative expression of *Cdkn2c* mRNA after siRNA treatment. siRNA2 significantly reduced the p18^INK4c^ mRNA expression level in *Bmi1*^*−/−*^ cochlear explants. **H** The images showed typical representations of spheres across WT, *Bmi1*^*−/−*^ and siRNA2 treated *Bmi1*^*−/−*^ groups. **I** The quantification of sphere areas revealed that the spheres in the *Bmi1*^*−/−*^ group were significantly smaller compared to those in the WT group. However, the introduction of siRNA2 notably increased the sphere area in the *Bmi1*^*−/−*^ group. **J** Immunofluorescence staining showing the Sox2 + cells, EdU + cells and Myo7a + cells of spheres in WT, *Bmi1*^*−/−*^ and siRNA treated *Bmi1*^*−/−*^ group. **K** The quantification of Sox2 + and EdU + cells within the spheres demonstrated that the numbers of both Sox2 + and EdU + cells in the *Bmi1*^*−/−*^ group were significantly lower compared to the WT group. However, treatment with siRNA2 notably increased the numbers of Sox2 + and EdU + cells in the *Bmi1*^*−/−*^ group. *p < 0.05, **p < 0.01, ***p < 0.001, ****p < 0.0001. Data shown as mean ± SEM. "n" represents the number of biological replicates analyzed. Scale bars were shown in sub-figures

To substantiate the observed changes, we applied a combination of immunohistochemical fluorescence staining, western blotting, and real-time qPCR to assess the protein and RNA expression levels of *Cdkn2c* in Bmi1 knockout mice. The western blotting, and real-time qPCR results indicated a notable increase in p18^INK4c^ expression, the protein product of *Cdkn2c*, within the sensory epithelial cells of neonatal *Bmi1*^−/−^ mice (Fig. 6A–C). The fluorescence intensity of p18^INK4c^ exhibited a gradient increase from the apical turn to the basal turn in both WT and *Bmi1*^−/−^ groups. Compared to the WT group, the *Bmi1*^−/−^ group showed significantly elevated p18 fluorescence intensity throughout the cochlear turns (Fig. 6D–F).

Following the observed upregulation of p18^INK4c^ in *Bmi1*^*−/−*^ mice, we employed siRNA technology to specifically reduce *Cdkn2c* expression. Initially, epithelial cells from *Bmi1*^−/−^ cochleae of neonatal mice were cultured in suspension. After a 7-day suspension culture, three specific siRNAs targeting *Cdkn2c*, along with a control siRNA, were introduced for 24 h. Among these, siRNA2 should the highest efficacy in knocking down *Cdkn2c*, as determined by RT-qPCR (Fig. 6G).

Subsequent experiment focused on sensory epithelial cells derived from WT and *Bmi1*^−/−^ cochleae, also cultured in suspension. For the *Bmi1*^−/−^ group, cultures were treated with *Cdkn2c* siRNA2. After another seven-day culture period, we observed significant differences in the morphology of cell aggregates. Notably, *Bmi1*^−/−^ group initially showed smaller sphere sizes compared to WT, but treatment with *Cdkn2c* siRNA led to a considerable increase in the size of these aggregates (Fig. 6H and I).

The experiments continued by transitioning these spheres to adherent culture conditions for five additional days. During this phase, EdU was incorporated to label proliferating cells. Quantitative analysis indicated a reduction in the number of EdU + cells within the *Bmi1*^−/−^ group relative to WT. However, *Cdkn2c* siRNA treatment resulted in a pronounced increase in EdU + cell counts, suggesting enhanced proliferative capacity. Additionally, we assessed the expression of the pluripotency marker Sox2 within these spheres. A significant decline in Sox2 + cells was noted in untreated *Bmi1*^−/−^ group compared to WT, but this decline was substantially reversed with *Cdkn2c* siRNA treatment, indicating a restoration of cell pluripotency (Fig. 6H and J). These experiments demonstrate that inhibition of *Cdkn2c* can counteract the proliferation deficits observed in *Bmi1*^−/−^ cochlear epithelial cells, thereby highlighting a potential therapeutic target for enhancing cell proliferation and regeneration in the sensory epithelium of the cochlea.

## Discussion

This study aimed to explore the role of *Bmi1* in the epigenetic regulation of cell fate within the auditory sensory epithelium. Despite its critical function in hearing and balance, the inner ear is a tiny organ with a limited number of hair cells and supporting cells, making it challenging to collect sufficient material for epigenetic analysis, even with advanced micro-volume techniques. Our study addresses this challenge by integrating ChIP-seq, ATAC-seq, and RNA-seq to highlight *Bmi1*’s pivotal role as an epigenetic regulator in the proliferation of otic progenitor cells. Among the genes identified, *Cdkn2c* (p18^INK4c^) emerged as a key player, and its suppression via siRNA in *Bmi1*^−/−^ mice significantly enhanced the proliferative capacity of sensory epithelial cells.

Bmi1 is well-established as a crucial regulator of cell proliferation. Previous studies, including our own, have demonstrated that *Bmi1* regulates the proliferation of progenitor and supporting cells in the neonatal mouse cochlea [22]. In this study, the absence of Bmi1 led to significant alterations in the expression of genes important for ear development and morphogenesis. Using a *Bmi1*^−/−^ mouse model, we elucidated Bmi1's role in H3K27me3-mediated epigenetic regulation of the inner ear. Auditory sensory epithelial cells from *Bmi1*^−/−^ mice exhibited significantly fewer and smaller spheres in suspension culture, and fewer EdU + cells in adherent culture compared to controls. However, there was no significant difference in the number of Myosin VIIa + cells per colony, suggesting that while Bmi1 knockout reduces cell proliferation, it does not impair the differentiation of supporting cells into hair cells. Adult *Bmi1*^−/−^ mice displayed hearing loss and sporadic hair cell loss, indicating that Bmi1 is essential for maintaining auditory function, likely by supporting the self-renewal capacity of inner ear supporting cells.

A key finding of this study is the identification of p18^INK4c^ as a regulator of cell proliferation, mediated by Bmi1 in the inner ear. Historically, Bmi1 has been recognized for its role in regulating stem cell self-renewal and differentiation, critical processes for tissue development [37, 38]. This regulation primarily occurs through the repression of the INK4a/Arf locus, which encodes p16^INK4a^ and p19^Arf^, both key regulators of the cell cycle and senescence [39]. In stem cells, downregulation of p16^INK4a^ and p19^Arf^ by Bmi1 promotes proliferation and prevents premature senescence, thereby maintaining the stem cell population [40]. Here, we found that the RNA expression level of *Cdkn2c*, encoding p18^INK4c^, was significantly upregulated in *Bmi1*^−/−^ mice, and this increase was accompanied by a reduction in H3K27me3 marks across the *Cdkn2c* gene. p18^INK4c^ is a member of the INK4 family of cyclin-dependent kinase (CDK) inhibitors [41], which inhibit CDK4 and CDK6 [42], thereby preventing retinoblastoma (Rb) protein phosphorylation and causing cell cycle arrest in the G1 phase[43]. Bmi1-mediated repression involves histone modifications, particularly the ubiquitination of histone H2A, which leads to chromatin condensation and transcriptional silencing of specific genes including the INK4a/ARF locus [44], where p18^INK4c^ is encoded. Given that *Cdkn2c* is a known tumor-suppressor gene that regulates cell proliferation[45], the decreased proliferation observed in inner ear progenitor cells following Bmi1 knockout might be attributed to the increased expression of p18^INK4c^ due to the decrease in H3K27me3 levels. Targeting the Bmi1- p18^INK4c^ pathway holds potential for therapeutic interventions in regenerative medicine [46, 47]. Strategies to transiently suppress p18^INK4c^ in aging tissues could rejuvenate stem cells and enhance tissue repair and regeneration. By using siRNA to suppress p18^INK4c^ expression, we observed a marked enhancement in the proliferative capacity of cochlear sensory epithelial cells in *Bmi1*^−/−^ mice. These results indicate that increased *Cdkn2c* expression of cochlear sensory epithelium cells in *Bmi1*^−/−^ mice could result to the reducing proliferating ability of sensory epithelial cells.

The development of inner ear and cell fate determination is driven by intricate gene expression patterns significantly influenced by epigenetic modifications such as histone acetylation and methylation, which are pivotal for toggling genes on and off during the developmental phases [48, 49]. In this study, RNA-seq data showed that in the sensory epithelial cells of *Bmi1*^*−/−*^ neonatal mouse inner ears, the transcriptome mRNA expression profile changes were mainly characterized by an upregulation of most genes (498 upregulated genes vs 53 downregulated genes). Epigenetic modifications facilitated by the PRC1 complex are known to result in gene silencing [50], changes in chromosome condensation, and chromosome remodeling [51–53]. Using ATAC-seq, this study examined the chromatin accessibility of cochlear sensory epithelial cells in *Bmi1*^*−/−*^ mice. The results indicated that there was a significant increase in chromatin accessibility in these cells (434 sites of increased accessibility vs 47 decreased). PcG proteins play a crucial role in this regulatory framework by modulating genes essential for cell lineage specification via histone methylation [54, 55]. As a member of PcG family, Bmi1 is integral to various biological processes including proliferation, self-renewal, migration, and differentiation of progenitor cells [56–58], and is also involved in modulating the levels of H3K27me3, primarily mediated by the PRC2 [31, 59–61]. Research by Gao et al. on early mouse embryonic development demonstrated the association of H3K27me3 with gene silencing, contrasting with H3K4me3, which is linked to gene activation [62]. Our current ChIP sequencing analysis further supports this, showing a notable decrease in H3K27me3 signals in the genome of *Bmi1*^−/−^ mice, accompanied by the upregulation of 498 genes, thus reinforcing the link between H3K27me3 modification and gene silencing (34 sites with increased H3K27me3 vs. 172 sites with decreased H3K27me3).

Through the collaborative actions of PRC1 and PRC2, Bmi1 plays a crucial role in repressing genes that are vital for differentiation processes. *Pax2*, a transcription factor critical for the development of the kidneys, eyes, ears, and central nervous system, is a prime example [50, 63–65]. Expression of *Pax2* needs precise regulation; both its up-expression and down-expression are associated with developmental anomalies and diseases [66]. Our data reveal significant enrichment peaks of H3K27me3 in the upstream regions of *Pax2*, suggesting that in tissues where *Pax2* should be inactive, Bmi1 and PRC2 collaboratively ensure its silencing to prevent inappropriate developmental signaling [67]. Conversely, during development, Pax2 expression is finely tuned in regions such as the kidney or midbrain [68, 69]. The removal or reduction of H3K27me3 marks by demethylases, which may be influenced by signals that also regulate Bmi1 levels, enables the activation of *Pax2* at appropriate locations and times [69]. Furthermore, enhancing H3K27me3 or *Bmi1* expression can help deactivate *Pax2* when its developmental role is complete, contributing to the precise temporal control of developmental processes [67].

Our study also acknowledges several limitations. We were unable to perform ChIP-seq on individual mice because the yield of inner ear sensory cells per animal was too low. To overcome this, we pooled samples over time to obtain sufficient material for epigenomic profiling. In addition, while our multi-omics approach identified *Cdkn2c* as a potential downstream mediator of *Bmi1*, and we showed that inhibition of *Cdkn2c* can partially compensate for *Bmi1* loss, we lack direct proof that Bmi1 binds and represses Cdkn2c through H3K27me3.

In conclusion, this study extends our understanding of how Bmi1 regulates the proliferation of sensory epithelial cells in the inner ear of neonatal mice. Our findings demonstrate that Bmi1 influences gene expression by modulating chromatin accessibility and H3K27me3 modifications. Future research is necessary to further elucidate the epigenetic landscape of the inner ear across different developmental stages and in disease contexts. Such investigations are essential for identifying specific epigenetic modifications linked to distinct phenotypic outcomes. Moreover, there is an urgent need to develop safe and effective methods for delivering epigenetic or gene therapies to the inner ear, which could hold significant therapeutic promise for treating hearing loss.

## Supplementary Information

Additional file 1.

## Data Availability

The study dataset and full trial protocol is available from the corresponding author upon reasonable request.

## References

[1] Wagner EL, Shin JB. Mechanisms of hair cell damage and repair. Trends Neurosci. 2019;42(6):414–24. 30992136 10.1016/j.tins.2019.03.006 PMC6556399

[2] Chan HL, Morey L. Emerging roles for polycomb-group proteins in stem cells and cancer. Trends Biochem Sci. 2019;44(8):688–700. 31085088 10.1016/j.tibs.2019.04.005

[3] Blackledge NP, Fursova NA, Kelley JR, Huseyin MK, Feldmann A, Klose RJ. PRC1 catalytic activity is central to polycomb system function. Mol Cell. 2020;77(4):857-874 e859. 31883950 10.1016/j.molcel.2019.12.001 PMC7033600

[4] Dobrinic P, Szczurek AT, Klose RJ. PRC1 drives polycomb-mediated gene repression by controlling transcription initiation and burst frequency. Nat Struct Mol Biol. 2021;28(10):811–24. 34608337 10.1038/s41594-021-00661-y PMC7612713

[5] Blackledge NP, Klose RJ. The molecular principles of gene regulation by Polycomb repressive complexes. Nat Rev Mol Cell Biol. 2021;22(12):815–33. 34400841 10.1038/s41580-021-00398-y PMC7612013

[6] Laugesen A, Hojfeldt JW, Helin K. Molecular mechanisms directing PRC2 recruitment and H3K27 methylation. Mol Cell. 2019;74(1):8–18. 30951652 10.1016/j.molcel.2019.03.011 PMC6452890

[7] Liu X, Wei W, Li X, Shen P, Ju D, Wang Z, et al. BMI1 and MEL18 promote colitis-associated cancer in mice via REG3B and STAT3. Gastroenterology. 2017;153(6):1607–20. 28780076 10.1053/j.gastro.2017.07.044

[8] Jia L, Zhang W, Wang CY. Bmi1 inhibition eliminates residual cancer stem cells after PD1 blockade and activates antitumor immunity to prevent metastasis and relapse. Cell Stem Cell. 2020;27(2):238-253.e236. 32697949 10.1016/j.stem.2020.06.022 PMC7416748

[9] Yang D, Liu HQ, Yang Z, Fan D, Tang QZ. BMI1 in the heart: novel functions beyond tumorigenesis. EBioMedicine. 2021;63:103193. 33421944 10.1016/j.ebiom.2020.103193 PMC7804972

[10] Kraus L, Bryan C, Wagner M, Kino T, Gunchenko M, Jalal W, et al. Bmi1 augments proliferation and survival of cortical bone-derived stem cells after injury through novel epigenetic signaling via histone 3 regulation. Int J Mol Sci. 2021. 10.3390/ijms22157813. 34360579 10.3390/ijms22157813 PMC8345961

[11] Hu T, Kitano A, Luu V, Dawson B, Hoegenauer KA, Lee BH, et al. Bmi1 suppresses adipogenesis in the hematopoietic stem cell niche. Stem Cell Reports. 2019;13(3):545–58. 31257132 10.1016/j.stemcr.2019.05.027 PMC6739622

[12] Kallin EM, Cao R, Jothi R, Xia K, Cui K, Zhao K, et al. Genome-wide uH2A localization analysis highlights Bmi1-dependent deposition of the mark at repressed genes. PLoS Genet. 2009;5(6):e1000506. 19503595 10.1371/journal.pgen.1000506 PMC2683938

[13] Cho YJ, Kim SH, Kim EK, Han JW, Shin KH, Hu H, et al. Prognostic implications of polycomb proteins ezh2, suz12, and eed1 and histone modification by H3K27me3 in sarcoma. BMC Cancer. 2018;18(1):158. 29415665 10.1186/s12885-018-4066-6 PMC5804074

[14] Choi R, Kurtenbach S, Goldstein BJ. Loss of BMI1 in mature olfactory sensory neurons leads to increased olfactory basal cell proliferation. Int Forum Allergy Rhinol. 2019;9(9):993–9. 31251849 10.1002/alr.22366 PMC7245364

[15] Lopez-Arribillaga E, Rodilla V, Pellegrinet L, Guiu J, Iglesias M, Roman AC, et al. Bmi1 regulates murine intestinal stem cell proliferation and self-renewal downstream of Notch. Development. 2015;142(1):41–50. 25480918 10.1242/dev.107714

[16] Biehs B, Hu JK, Strauli NB, Sangiorgi E, Jung H, Heber RP, et al. BMI1 represses Ink4a/Arf and Hox genes to regulate stem cells in the rodent incisor. Nat Cell Biol. 2013;15(7):846–52. 23728424 10.1038/ncb2766 PMC3735916

[17] Akala OO, Clarke MF. Hematopoietic stem cell self-renewal. Curr Opin Genet Dev. 2006;16(5):496–501. 16919448 10.1016/j.gde.2006.08.011

[18] Bordeleau ME, Aucagne R, Chagraoui J, Girard S, Mayotte N, Bonneil E, et al. UBAP2L is a novel BMI1-interacting protein essential for hematopoietic stem cell activity. Blood. 2014;124(15):2362–9. 25185265 10.1182/blood-2014-01-548651 PMC4192749

[19] Liu S, Wu M, Lancelot M, Deng J, Gao Y, Roback JD, et al. BMI1 enables extensive expansion of functional erythroblasts from human peripheral blood mononuclear cells. Mol Ther. 2021;29(5):1918–32. 33484967 10.1016/j.ymthe.2021.01.022 PMC8116606

[20] Vora P, Seyfrid M, Venugopal C, Qazi MA, Salim S, Isserlin R, et al. Bmi1 regulates human glioblastoma stem cells through activation of differential gene networks in CD133+ brain tumor initiating cells. J Neurooncol. 2019;143(3):417–28. 31115870 10.1007/s11060-019-03192-1

[21] Yadirgi G, Leinster V, Acquati S, Bhagat H, Shakhova O, Marino S. Conditional activation of Bmi1 expression regulates self-renewal, apoptosis, and differentiation of neural stem/progenitor cells in vitro and in vivo. Stem Cells. 2011;29(4):700–12. 21305672 10.1002/stem.614

[22] Lu X, Sun S, Qi J, Li W, Liu L, Zhang Y, et al. Bmi1 regulates the proliferation of cochlear supporting cells via the canonical Wnt signaling pathway. Mol Neurobiol. 2017;54(2):1326–39. 26843109 10.1007/s12035-016-9686-8

[23] van der Lugt NM, Domen J, Linders K, van Roon M, Robanus-Maandag E, te Riele H, et al. Posterior transformation, neurological abnormalities, and severe hematopoietic defects in mice with a targeted deletion of the bmi-1 proto-oncogene. Genes Dev. 1994;8(7):757–69. 7926765 10.1101/gad.8.7.757

[24] Anders S, Pyl PT, Huber W. HTSeq–a Python framework to work with high-throughput sequencing data. Bioinformatics. 2015;31(2):166–9. 25260700 10.1093/bioinformatics/btu638 PMC4287950

[25] Love MI, Huber W, Anders S. Moderated estimation of fold change and dispersion for RNA-seq data with DESeq2. Genome Biol. 2014;15(12):550. 25516281 10.1186/s13059-014-0550-8 PMC4302049

[26] Bolger AM, Lohse M, Usadel B. Trimmomatic: a flexible trimmer for Illumina sequence data. Bioinformatics. 2014;30(15):2114–20. 24695404 10.1093/bioinformatics/btu170 PMC4103590

[27] Koehler KR, Nie J, Longworth-Mills E, Liu XP, Lee J, Holt JR, et al. Generation of inner ear organoids containing functional hair cells from human pluripotent stem cells. Nat Biotechnol. 2017;35(6):583–9. 28459451 10.1038/nbt.3840 PMC5462862

[28] Mittal R, Nguyen D, Patel AP, Debs LH, Mittal J, Yan D, Eshraghi AA, Van De Water TR, Liu XZ: Recent advancements in the regeneration of auditory hair cells and hearing restoration. Front Mol Neurosci 2017; 10:236 10.3389/fnmol.2017.00236 PMC5534485 28824370

[29] Czajkowski A, Mounier A, Delacroix L, Malgrange B. Pluripotent stem cell-derived cochlear cells: a challenge in constant progress. Cell Mol Life Sci. 2019;76(4):627–35. 30341460 10.1007/s00018-018-2950-5 PMC11105202

[30] Sun S, Li S, Luo Z, Ren M, He S, Wang G, et al. Dual expression of Atoh1 and Ikzf2 promotes transformation of adult cochlear supporting cells into outer hair cells. Elife. 2021. 10.7554/eLife.66547. 34477109 10.7554/eLife.66547 PMC8439656

[31] Kushwaha AC, Mohanbhai SJ, Sardoiwala MN, Sood A, Karmakar S, Roy Choudhury S. Epigenetic regulation of Bmi1 by ubiquitination and proteasomal degradation inhibit Bcl-2 in acute myeloid leukemia. ACS Appl Mater Interfaces. 2020;12(23):25633–44. 32453568 10.1021/acsami.0c06186

[32] Benard A, Goossens-Beumer IJ, van Hoesel AQ, Horati H, Putter H, Zeestraten EC, et al. Prognostic value of polycomb proteins EZH2, BMI1 and SUZ12 and histone modification H3K27me3 in colorectal cancer. PLoS ONE. 2014;9(9):e108265. 25243792 10.1371/journal.pone.0108265 PMC4171510

[33] Cai Y, Zhang Y, Loh YP, Tng JQ, Lim MC, Cao Z, et al. H3K27me3-rich genomic regions can function as silencers to repress gene expression via chromatin interactions. Nat Commun. 2021;12(1):719. 33514712 10.1038/s41467-021-20940-y PMC7846766

[34] Sanchez A, De Vivo A, Uprety N, Kim J, Stevens SM Jr, Kee Y. BMI1-UBR5 axis regulates transcriptional repression at damaged chromatin. Proc Natl Acad Sci U S A. 2016;113(40):11243–8. 27647897 10.1073/pnas.1610735113 PMC5056041

[35] Grandi FC, Modi H, Kampman L, Corces MR. Chromatin accessibility profiling by ATAC-seq. Nat Protoc. 2022;17(6):1518–52. 35478247 10.1038/s41596-022-00692-9 PMC9189070

[36] Guo Y, Zhao S, Wang GG. Polycomb gene silencing mechanisms: PRC2 chromatin targeting, H3K27me3 “readout”, and phase separation-based compaction. Trends Genet. 2021;37(6):547–65. 33494958 10.1016/j.tig.2020.12.006 PMC8119337

[37] Lukacs RU, Memarzadeh S, Wu H, Witte ON. Bmi-1 is a crucial regulator of prostate stem cell self-renewal and malignant transformation. Cell Stem Cell. 2010;7(6):682–93. 21112563 10.1016/j.stem.2010.11.013 PMC3019762

[38] Siddique HR, Saleem M. Role of BMI1, a stem cell factor, in cancer recurrence and chemoresistance: preclinical and clinical evidences. Stem Cells. 2012;30(3):372–8. 22252887 10.1002/stem.1035

[39] Jacobs JJ, Kieboom K, Marino S, DePinho RA, Van Lohuizen M. The oncogene and Polycomb-group gene bmi-1 regulates cell proliferation and senescence through the ink4a locus. Nature. 1999;397(6715):164–8. 9923679 10.1038/16476

[40] Chen G, Zhang Y, Yu S, Sun W, Miao D. Bmi1 overexpression in mesenchymal stem cells exerts antiaging and antiosteoporosis effects by inactivating p16/p19 signaling and inhibiting oxidative stress. Stem Cells. 2019;37(9):1200–11. 30895687 10.1002/stem.3007 PMC6851636

[41] Hirai H, Roussel MF, Kato J-Y, Ashmun RA, Sherr CJ. Novel INK4 proteins, p19 and p18, are specific inhibitors of the cyclin D-dependent kinases CDK4 and CDK6. Mol Cell Biol. 1995. 10.1128/MCB.15.5.2672. 7739547 10.1128/mcb.15.5.2672 PMC230497

[42] Zhu S, Cao J, Sun H, Liu K, Li Y, Zhao T. P18 inhibits reprogramming through inactivation of Cdk4/6. Sci Rep. 2016;6(1):31085. 27484146 10.1038/srep31085 PMC4971472

[43] Guan K-L, Jenkins CW, Li Y, Nichols MA, Wu X, O’Keefe CL, et al. Growth suppression by p18, a p16INK4/MTS1-and p14INK4B/MTS2-related CDK6 inhibitor, correlates with wild-type pRb function. Genes Dev. 1994;8(24):2939–52. 8001816 10.1101/gad.8.24.2939

[44] Ginjala V, Nacerddine K, Kulkarni A, Oza J, Hill SJ, Yao M, et al. BMI1 is recruited to DNA breaks and contributes to DNA damage-induced H2A ubiquitination and repair. Mol Cell Biol. 2011;31(10):1972–82. 21383063 10.1128/MCB.00981-10 PMC3133356

[45] Cheng J, Demeulemeester J, Wedge DC, Vollan HKM, Pitt JJ, Russnes HG, et al. Pan-cancer analysis of homozygous deletions in primary tumours uncovers rare tumour suppressors. Nat Commun. 2017;8(1):1221. 29089486 10.1038/s41467-017-01355-0 PMC5663922

[46] Yang M, Tang Y, Zhu P, Lu H, Wan X, Guo Q, et al. The advances of E2A-PBX1 fusion in B-cell acute lymphoblastic leukaemia. Ann Hematol. 2023. 10.1007/s00277-023-05595-7. 38148344 10.1007/s00277-023-05595-7

[47] Malakoti F, Alemi F, Younesi S, Majidinia M, Yousefi B, Morovat P, et al. The cross-talk between signaling pathways, noncoding RNAs and DNA damage response: emerging players in cancer progression. DNA Repair. 2021;98:103036. 33429260 10.1016/j.dnarep.2020.103036

[48] Provenzano MJ, Domann FE. A role for epigenetics in hearing: establishment and maintenance of auditory specific gene expression patterns. Hear Res. 2007;233(1–2):1–13. 17723285 10.1016/j.heares.2007.07.002 PMC2994318

[49] Jambhekar A, Dhall A, Shi Y. Roles and regulation of histone methylation in animal development. Nat Rev Mol Cell Biol. 2019;20(10):625–41. 31267065 10.1038/s41580-019-0151-1 PMC6774358

[50] Bosze B, Suarez-Navarro J, Soofi A, Lauderdale JD, Dressler GR, Brown NL. Multiple roles for Pax2 in the embryonic mouse eye. Dev Biol. 2021;472:18–29. 33428890 10.1016/j.ydbio.2020.12.020 PMC7956245

[51] Yokoyama Y, Arai MA, Hara Y, Ishibashi M. Identification of BMI1 promoter inhibitors from *Streptomyces* sp. IFM-11958. Bioorg Med Chem. 2019;27(13):2998–3003. 31079965 10.1016/j.bmc.2019.05.002

[52] Eeftens JM, Kapoor M, Michieletto D, Brangwynne CP. Polycomb condensates can promote epigenetic marks but are not required for sustained chromatin compaction. Nat Commun. 2021;12(1):5888. 34620850 10.1038/s41467-021-26147-5 PMC8497513

[53] Roule T, Christ A, Hussain N, Huang Y, Hartmann C, Benhamed M, et al. The lncRNA MARS modulates the epigenetic reprogramming of the marneral cluster in response to ABA. Mol Plant. 2022;15(5):840–56. 35150931 10.1016/j.molp.2022.02.007

[54] Aranda S, Mas G, Di Croce L. Regulation of gene transcription by Polycomb proteins. Sci Adv. 2015;1(11):e1500737. 26665172 10.1126/sciadv.1500737 PMC4672759

[55] Loubiere V, Martinez AM, Cavalli G. Cell fate and developmental regulation dynamics by polycomb proteins and 3D genome architecture. BioEssays. 2019;41(3):1800222. 10.1002/bies.201800222 30793782

[56] Yu H, Gao R, Chen S, Liu X, Wang Q, Cai W, et al. Bmi1 regulates Wnt signaling in hematopoietic stem and progenitor cells. Stem Cell Rev Rep. 2021;17:2304–13. 34561772 10.1007/s12015-021-10253-4 PMC9097559

[57] Ganapathi M, Boles NC, Charniga C, Lotz S, Campbell M, Temple S, et al. Effect of Bmi1 over-expression on gene expression in adult and embryonic murine neural stem cells. Sci Rep. 2018;8(1):7464. 29749381 10.1038/s41598-018-25921-8 PMC5945652

[58] Kato Y, Hou L-B, Miyagi S, Nitta E, Aoyama K, Shinoda D, et al. Bmi1 restricts the adipogenic differentiation of bone marrow stromal cells to maintain the integrity of the hematopoietic stem cell niche. Exp Hematol. 2019;76:24–37. 31408689 10.1016/j.exphem.2019.07.006

[59] Ghosh K, Chatterjee B, Maheswari U, Athifa M, Kanade SR. 4-nonylphenol-enhanced EZH2 and RNF2 expression, H3K27me3 and H2AK119ub1 marks resulting in silencing of p21(CDKN1A) *in vitro*. Epigenomics. 2019;11(8):899–916. 31144530 10.2217/epi-2018-0175

[60] Kumar A, Kondhare KR, Vetal PV, Banerjee AK. Pcg proteins MSI1 and BMI1 function upstream of miR156 to regulate aerial tuber formation in potato. Plant Physiol. 2020;182(1):185–203. 31427464 10.1104/pp.19.00416 PMC6945842

[61] Ricci B, Millner TO, Pomella N, Zhang X, Guglielmi L, Badodi S, et al. Polycomb-mediated repression of EphrinA5 promotes growth and invasion of glioblastoma. Oncogene. 2020;39(12):2523–38. 31988455 10.1038/s41388-020-1161-3 PMC7082224

[62] Liu X, Wang C, Liu W, Li J, Li C, Kou X, et al. Distinct features of H3K4me3 and H3K27me3 chromatin domains in pre-implantation embryos. Nature. 2016;537(7621):558–62. 27626379 10.1038/nature19362

[63] Lv N, Wang Y, Zhao M, Dong L, Wei H. The role of PAX2 in neurodevelopment and disease. Neuropsychiatr Dis Treat. 2021. 10.2147/NDT.S332747. 34908837 10.2147/NDT.S332747 PMC8665868

[64] Harshman LA, Brophy PD. PAX2 in human kidney malformations and disease. Pediatr Nephrol. 2012;27:1265–75. 22138676 10.1007/s00467-011-2053-0

[65] Burton Q, Cole LK, Mulheisen M, Chang W, Wu DK. The role of Pax2 in mouse inner ear development. Dev Biol. 2004;272(1):161–75. 15242798 10.1016/j.ydbio.2004.04.024

[66] Bower MA, Schimmenti LA, Eccles MR: PAX2-related disorder. 2018. 20301624

[67] Zhou Y, Wang L, Vaseghi HR, Liu Z, Lu R, Alimohamadi S, et al. Bmi1 is a key epigenetic barrier to direct cardiac reprogramming. Cell Stem Cell. 2016;18(3):382–95. 26942853 10.1016/j.stem.2016.02.003 PMC4779178

[68] Dressler GR, Patel SR. Epigenetics in kidney development and renal disease. Transl Res. 2015;165(1):166–76. 24958601 10.1016/j.trsl.2014.04.007 PMC4256142

[69] Feng X, Juan AH, Wang HA, Ko KD, Zare H, Sartorelli V. Polycomb Ezh2 controls the fate of GABAergic neurons in the embryonic cerebellum. Development. 2016;143(11):1971–80. 27068104 10.1242/dev.132902 PMC4920161

