# Supplementary material for: Bmi1 controls auditory sensory epithelial cell proliferation through genome-wide H3K27me3 modifications
Source: Epigenetics Chromatin. 2025 Nov 19;18:75. doi: 10.1186/s13072-025-00642-1 (PMC12629052; doi:10.1186/s13072-025-00642-1)
Supplement: Supplementary file 1 — Additional file 1. [file 13072_2025_642_MOESM1_ESM.docx]

**Supplementary figure legends**.

**
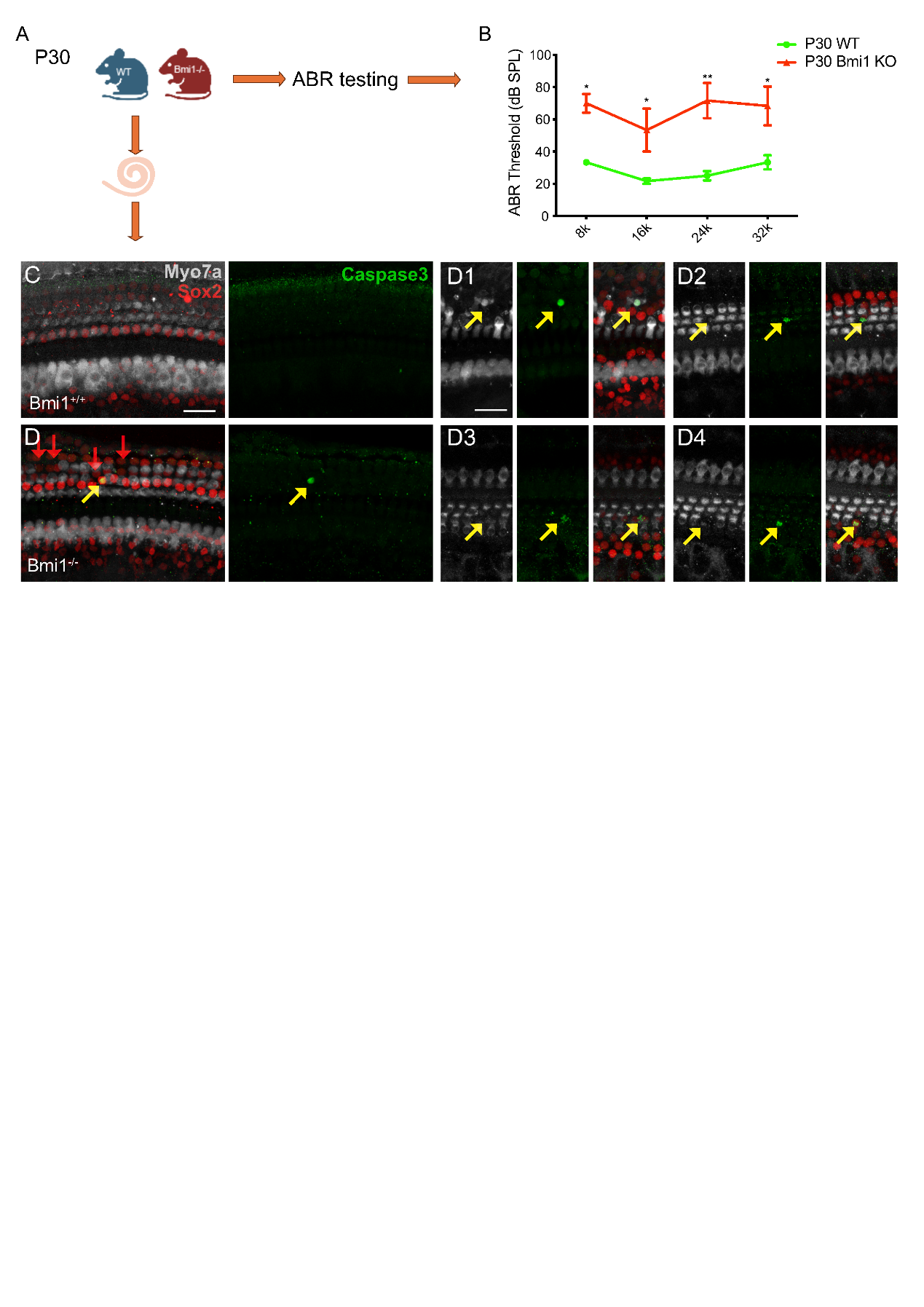
**

**Figure S1: Bmi1 gene knockout caused hearing loss and hair cell apoptosis in mice.** (A) A schematic diagram illustrating auditory brainstem response (ABR) testing in WT and *Bmi1^-/-^* mice at P30, followed by immunohistochemical staining of the inner ear's organ of Corti. (B) The ABR results indicate that the threshold in *Bmi1^-/-^* mice was significantly higher across frequencies of 8, 16, 24, and 32 kHz than that in the WT group. The sound intensity is displayed in decibels sound pressure level (dB SPL). (C) No caspase-3+ hair cell was found in the OC of WT mice. (D, D1-4) Caspase-3+ hair cells were observed in the OC of *Bmi1^-/-^* mice. Red arrows indicate missing hair cells, while yellow arrows denote Caspase-3^+^ cells. Data are presented as Mean ± S.E.M. **p* < 0.05, ***p* < 0.01.


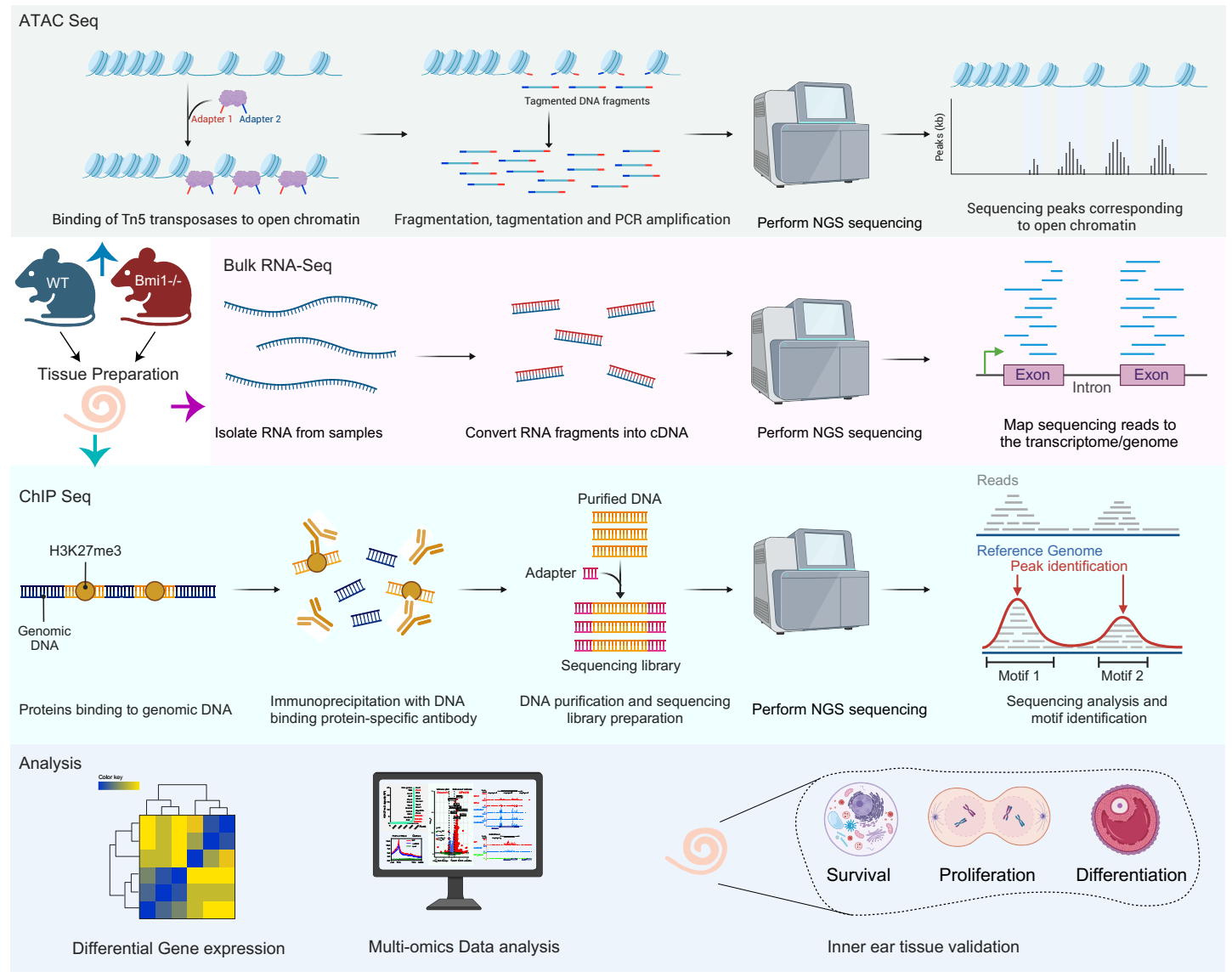


**Figure S2**: A flowchart of RNA-seq, ChIP-seq, and ATAC-seq for sensory epithelial cells of the mouse inner ear.

**Supplementary table 1: Reagent and resource.**

| **REAGENT or RESOURCE** | | **SOURCE** | **IDENTIFIER** |
| --- | --- | --- | --- |
| **Antibodies** | | | |
| Rabbit anti-Myosin VIIa | | Proteus Biosciences | Cat# 25-6790; AB_10015251 |
| Goat anti-Sox2 | | Santa Cruz Biotechnology | Cat# sc-17320; AB_2286684 |
| Mouse anti-p18^INK4C^ | | Santa Cruz Biotechnology | Cat# sc-9965; |
| Mouse anti-Histone H3 (tri methyl K27) | | Abcam | Cat# ab6002; AB_305237; |
| Rabbit anti-Caspase3 | | Abcam | Cat# ab32351; AB_725946; |
| GAPDH Rabbit Antibody (HRP conjugated) | | Abmart | Cat# T40004; AB_2936279; |
| DAPI | | Invitrogen | Cat# D1306; AB_2629482; |
| Alexa Fluor™ Donkey anti-mouse IgG 488 | | Jackson Immuno Research | Cat# 715545150; AB_2340846; |
| Alexa Fluor™ Donkey anti-rabbit IgG 594 | | Jackson Immuno Research | Cat# 711585152; AB_2340621; |
| Alexa Fluor™ Donkey anti-goat IgG 647 | | Jackson Immuno Research | Cat# 705605003; AB_2340436; |
| Goat Anti-Rabbit IgG-HRP | | Abmart | Cat# M21002; AB_2713951; |
| Goat Anti-Mouse IgG- HRP | | Abmart | Cat# M21001; AB_2713950; |
| Click-iT™ EdU Cell Proliferation Kit for Imaging, Alexa Fluor™ 488 dye | | Thermo Fisher Scientific | Cat# C10337 |
| **Deposited data** | | | |
| ChIP sequencing | | Gene Expression Omnibus | GSE279539 |
| transcriptomic sequencing | | Gene Expression Omnibus | GSE279541 |
| ATAC sequencing | | Gene Expression Omnibus | GSE279758 |
| **Experimental models: Organisms/strains** | | | |
| ***Bmi1+/−* mice** | | *van der Lugt et al. Genes Dev. 1994* | [FVB.129P2-*Bmi1^tm1Brn^*/MvlJ](https://www.jax.org/strain/024584), Strain #:024584 |
| **Other** | | | |
| *Cdkn2c Primers* | F: GGGGACCTAGAGCAACTTACT | | R: AAATTGGGATTAGCACCTCTGAG |
| *Bmi1 Primers* | F: AAATCCCCACTTAATGTGTGTCC | | R: CTTGCTGGTCTCCAAGTAACG |
| *Gapdh Primers* | F: AGGTCGGTGTGAACGGATTTG | | R: GGGGTCGTTGATGGCAACA |
| mCdkn2c-siRNA2 siRNA *Primers* | Sense: GCAGGUUAUGAAACUUGGAAATT | | Antisense: UUUCCAAGUUUCAUAACCUGCTT |

**Supplementary table 2:** **Top 25 upregulated genes of RNA-seq by the *p* value with their function**

| **Top genes** | **baseMean** | **log_2_ FoldChange** | **pvalue** | **Function summary (****from NCBI Gene Summary)** |
| --- | --- | --- | --- | --- |
| Spag6 | 226.757748 | 4.0368083 | 9.08E-68 | Predicted to enable microtubule binding activity. |
| Tbx15 | 561.994593 | 4.06559362 | 1.47E-50 | Involved in negative regulation of transcription by RNA polymerase II. |
| Cacng5 | 1123.36253 | 1.28665209 | 9.49E-41 | Predicted to enable channel regulator activity and voltage-gated calcium channel activity. |
| Myh7b | 872.902869 | 2.02424345 | 4.67E-33 | This gene encodes a myosin heavy chain. |
| Trank1 | 677.930525 | 2.27862682 | 8.19E-26 | Is expressed in adrenal cortex; central nervous system; frenulum; lung; and seminiferous cord. Orthologous to human TRANK1 (tetratricopeptide repeat and ankyrin repeat containing 1). |
| Flt3 | 119.182334 | 2.63339933 | 4.23E-24 | Enables several functions, including phosphatidylinositol 3-kinase binding activity; protein tyrosine kinase activity; and ubiquitin protein ligase binding activity. Involved in several processes, including common myeloid progenitor cell proliferation; hemopoiesis; and lymphocyte proliferation. |
| Bhlhe22 | 533.950802 | 1.90647869 | 5.02E-24 | Enables DNA-binding transcription factor activity, RNA polymerase II-specific; chromatin binding activity; and identical protein binding activity. Acts upstream of or within cerebral cortex regionalization; negative regulation of DNA-templated transcription; and neuron differentiation. |
| Thy1 | 2538.35507 | 1.8589904 | 3.48E-21 | This gene encodes a glycoprotein that is anchored to the cell surface of thymocytes, neuronal and other cells through a glycosyl-phosphatidylinositol moiety. |
| Raet1e | 95.4755864 | 2.75424431 | 3.79E-21 | Enables natural killer cell lectin-like receptor binding activity. Acts upstream of or within T cell mediated cytotoxicity. |
| Nefl | 15286.9462 | 1.70062757 | 3.44E-20 | Enables protein-macromolecule adaptor activity. A structural constituent of postsynaptic intermediate filament cytoskeleton. |
| Cnpy1 | 84.9454116 | 4.52685836 | 4.88E-20 | Is expressed in several structures, including brain and olfactory epithelium. |
| Calb2 | 13032.0699 | 1.06477123 | 8.88E-20 | Enables calcium ion binding activity involved in regulation of presynaptic cytosolic calcium ion concentration. Predicted to be involved in regulation of presynaptic cytosolic calcium ion concentration. |
| Rgs7 | 465.72182 | 1.48227249 | 1.14E-19 | Enables GTPase activator activity. Involved in G protein-coupled receptor signaling pathway and regulation of postsynaptic membrane potential. |
| Hoxd8 | 85.6310036 | 2.81258743 | 1.52E-18 | Predicted to enable DNA-binding transcription activator activity, RNA polymerase II-specific and RNA polymerase II transcription regulatory region sequence-specific DNA binding activity. |
| Zic4 | 246.38402 | 2.04725121 | 2.46E-18 | Enables RNA polymerase II transcription regulatory region sequence-specific DNA binding activity. |
| Hcn4 | 1771.35538 | 0.88747552 | 3.77E-18 | Enables intracellularly cAMP-activated cation channel activity. |
| Gria4 | 4552.56421 | 0.71592679 | 2.18E-17 | Glutamate receptors are the predominant excitatory neurotransmitter receptors in the mammalian brain and are activated in a variety of normal neurophysiologic processes. |
| Nap1l5 | 2151.16026 | 1.53513662 | 2.27E-17 | Predicted to be involved in nucleosome assembly. |
| Galnt17 | 2134.01652 | 0.86064508 | 5.76E-17 | Predicted to enable polypeptide N-acetylgalactosaminyltransferase activity. |
| Reln | 893.700837 | 1.15377134 | 1.15E-16 | Enables receptor ligand activity; serine-type peptidase activity; and very-low-density lipoprotein particle receptor binding activity. |
| Cdkn2c | 1190.77961 | 1.28666197 | 1.76E-16 | The protein encoded by this gene is a member of the INK4 family of cyclin-dependent kinase (cdk) inhibitors, and contains five ankyrin repeats. |
| Kcnk9 | 4032.33881 | 1.76649271 | 2.54E-16 | Enables outward rectifier potassium channel activity and protein heterodimerization activity. |
| Sfmbt2 | 672.647347 | 0.98905679 | 4.62E-16 | Predicted to be involved in negative regulation of DNA-templated transcription and negative regulation of gene expression. |
| Hcn2 | 1043.15073 | 1.342742 | 5.18E-16 | Predicted to enable several functions, including PDZ domain binding activity; identical protein binding activity; and monoatomic cation channel activity. |
| Scn1a | 2383.61593 | 1.74310807 | 5.22E-16 | Enables voltage-gated sodium channel activity. |
